# The Trajectory of Dispersal Research in Conservation Biology. Systematic Review

**DOI:** 10.1371/journal.pone.0095053

**Published:** 2014-04-17

**Authors:** Don A. Driscoll, Sam C. Banks, Philip S. Barton, Karen Ikin, Pia Lentini, David B. Lindenmayer, Annabel L. Smith, Laurence E. Berry, Emma L. Burns, Amanda Edworthy, Maldwyn J. Evans, Rebecca Gibson, Rob Heinsohn, Brett Howland, Geoff Kay, Nicola Munro, Ben C. Scheele, Ingrid Stirnemann, Dejan Stojanovic, Nici Sweaney, Nélida R. Villaseñor, Martin J. Westgate

**Affiliations:** 1 ARC Centre of Excellence for Environmental Decisions, the NERP Environmental Decisions Hub, Fenner School of Environment and Society, The Australian National University, Canberra, Australian Capital Territory, Australia; 2 School of Botany, University of Melbourne, Melbourne, Victoria, Australia; 3 School of Biological Sciences, University of Wollongong, Wollongong, New South Wales, Australia; University of Waikato (National Institute of Water and Atmospheric Research), New Zealand

## Abstract

Dispersal knowledge is essential for conservation management, and demand is growing. But are we accumulating dispersal knowledge at a pace that can meet the demand? To answer this question we tested for changes in dispersal data collection and use over time. Our systematic review of 655 conservation-related publications compared five topics: climate change, habitat restoration, population viability analysis, land planning (systematic conservation planning) and invasive species. We analysed temporal changes in the: (i) questions asked by dispersal-related research; (ii) methods used to study dispersal; (iii) the quality of dispersal data; (iv) extent that dispersal knowledge is lacking, and; (v) likely consequences of limited dispersal knowledge. Research questions have changed little over time; the same problems examined in the 1990s are still being addressed. The most common methods used to study dispersal were occupancy data, expert opinion and modelling, which often provided indirect, low quality information about dispersal. Although use of genetics for estimating dispersal has increased, new ecological and genetic methods for measuring dispersal are not yet widely adopted. Almost half of the papers identified knowledge gaps related to dispersal. Limited dispersal knowledge often made it impossible to discover ecological processes or compromised conservation outcomes. The quality of dispersal data used in climate change research has increased since the 1990s. In comparison, restoration ecology inadequately addresses large-scale process, whilst the gap between knowledge accumulation and growth in applications may be increasing in land planning. To overcome apparent stagnation in collection and use of dispersal knowledge, researchers need to: (i) improve the quality of available data using new approaches; (ii) understand the complementarities of different methods and; (iii) define the value of different kinds of dispersal information for supporting management decisions. Ambitious, multi-disciplinary research programs studying many species are critical for advancing dispersal research.

## Introduction

Dispersal is a fundamental behavioural and ecological process that influences the distribution of biodiversity in every ecosystem [1]–. The distance that individuals disperse, and the number of dispersers can be the primary determinant of where and whether species persist [5], [6]. Dispersal fundamentally influences spatial population dynamics including metapopulation and metacommunity processes [7], [8]. For animals, the process of dispersing from a natal territory to find new space in which to live and avoid inbreeding strongly influences individual fitness [9], [10]. Individual fitness, in turn, impacts on the social and genetic structure of populations and their viability [11]–[13].

Because dispersal has such an important ecological role, knowledge of where and when species move is critical for managing and conserving biodiversity, especially in fragmented landscapes [14], [15]. Much has been learnt about dispersal, particularly from an evolutionary perspective [16]–[18], and the proportion of papers addressing movement (including dispersal, migration, home-range movements) increases by 0.3% each year [19]. Despite this, there is concern that knowledge of dispersal remains inadequate [20], [21]. Recent reviews of the most important unanswered questions in conservation management and policy reveal that better knowledge of dispersal is needed, principally in relation to improving connectivity and reversing habitat fragmentation [22]–[28]. Furthermore, uncertainty about how effectively restoration can improve connectivity and facilitate metapopulation dynamics has engendered debate about whether connectivity should be a conservation priority [29]–[31].

To what extent can we be optimistic that the rate of knowledge accumulation from dispersal research can keep up with problem identification in biodiversity conservation? On the one hand there has been substantial technological progress in measuring dispersal, including genetic and direct approaches [32], [33], so substantial changes in the quality and application of dispersal knowledge might be expected. On the other hand, it is not clear how widely these new techniques are applied. If new techniques are not widely applied and if the number of applications is expanding [34], the knowledge gap about dispersal may be getting bigger.

Our approach in this review was to examine *how* we learn about dispersal to gauge how the field has progressed and to help define areas where new directions may be needed. This is in contrast to previous reviews and books that focus on *what* we have learnt about dispersal [4], [35]–[38]. We first examined the scope of dispersal research by asking: (1) what research applications are addressed with dispersal data? To describe the extent that methodology may limit our understanding of dispersal, we asked: (2) which methods are used to collect dispersal data? We discuss the application of commonly applied methods for measuring dispersal, highlighting the strengths and limitations of the data that most studies use. We then examine five metrics summarising data quality to answer the question: (3) what is the quality of dispersal knowledge? Our fourth question was (4) to what extent is dispersal regarded as a knowledge gap? In addressing this question we compared dispersal with non-dispersal gaps in knowledge to understand how common dispersal knowledge gaps are relative to other gaps. To understand whether any dispersal-related knowledge gaps should be regarded as important we asked: (5) what would be the likely consequences for research and conservation if dispersal knowledge was not available? In addition, we assessed biases related to taxonomic group, biome and region of the globe in which the work was undertaken [39]. For each of the five questions, we were interested in the trajectory of dispersal research and therefore we compared research from two time periods (the 1990s and 2010-12) to determine if answers to our five questions changed over time.

A second focus of our review was on specific topics. This enabled us to discover whether some sub-disciplines within conservation biology are doing better than others with regard to the collection and application of dispersal data. Although there are many ways in which the field of conservation biology could be segregated into separate topics, we have chosen five in which dispersal knowledge is important and which are of global relevance. These are climate change, habitat restoration, population viability analysis (PVA), invasive species and land planning. These topics encompass the most serious threats to biodiversity, including climate change, invasive species, and habitat loss (the latter is addressed indirectly through the habitat restoration topic). These topics also include some widely used approaches to attempting to solve biodiversity management problems (restoration, land planning and PVA). Next, we briefly explain how dispersal knowledge is critical to each of these topics.

### Five topics in conservation that depend on dispersal knowledge

#### Climate change

Climate change is geographically shifting the climatic envelope of many species [40]–[43] and this could occur rapidly in some biomes (more than 1 km/yr [44]). Species' survival may therefore depend on the ability of individuals to disperse across the landscape, allowing populations to follow the shifting location of their realised niche [45]–[48]. Further, climate change is expected to interact with habitat fragmentation whereby reduced dispersal in fragmented landscapes reduces a species' capacity for range expansion in response to changing local climatic conditions [49], [50]. Knowledge of dispersal is therefore an essential component of predicting and ameliorating the effects of climate change on biodiversity [51]–[53].

#### Habitat restoration

Habitat restoration is widely advocated as a solution to degradation, fragmentation or loss of native vegetation [54]. Dispersal strongly influences the outcome of habitat restoration, with recolonisation of planted vegetation by a range of plants and animals a core assumption underlying the approach [55]–[57]. However, based on the Field of Dreams hypothesis [58] the extent to which ‘they will come, if we build it’ needs to be determined, along with a more detailed understanding about where they might come from, by what route, and under what environmental conditions [59]–[61].

#### Population viability analysis (PVA)

The aim of PVA is to understand the risk of extinction and typically uses stochastic models that describe population trajectories through time, often under contrasting management scenarios [62]. Conceptually, PVA is a suite of methods and is not a topic in the same sense as the other four topics. However, we included it in our review because these tools are frequently applied in conservation and depend on dispersal data. Population viability can be strongly influenced by dispersal [63] and thus PVA analyses often incorporate dispersal estimates to parameterise metapopulation simulations [64], [65]. The outputs of PVA simulations are sensitive to parameters related to how far and how often species disperse, and to mortality during dispersal [66]. Because of the importance of using reliable dispersal parameters, there are many examples in the PVA literature that indicate additional dispersal data are needed [67].

#### Land planning

Land planning, also called systematic conservation planning [68], includes studies that aim to prioritise regions for conservation or other activities, including prioritising the location of reserves and linking corridors [69]. Land planning and reserve design make extensive use of connectivity principles, often based on theory [70], [71], but require dispersal data to verify assumptions [72], [73]. For example, substantially different conservation priorities can emerge when corridor design considers the dispersal ability of animals through potential corridors compared with if they do not [74]. Good dispersal data are needed to support land-planning models and to make land-planning decisions that provide for movement of organisms across the landscape [75].

#### Invasive species

Invasive species are a major driver of ecosystem transformation and biodiversity loss [76], [77]. Dispersal knowledge is essential for understanding the capacity for management or biological controls to reduce invasive species threats [78], [79]. Understanding invasion risk also depends fundamentally on knowledge of the extent of dispersal through different kinds of landscapes [80], [81] and how dispersal is mediated by dispersal vectors [82], [83]. For example, using radio-tracking data for cane toads (*Rhinella marina*), Tingley et al. [84] modelled rates of invasion in NW Australia. They were able to identify key points where removing artificial water bodies would create a barrier to cane toad expansion, excluding them from 268,000 km^2^ of their potential range.

## Methods

### Database

**Figure 1:**
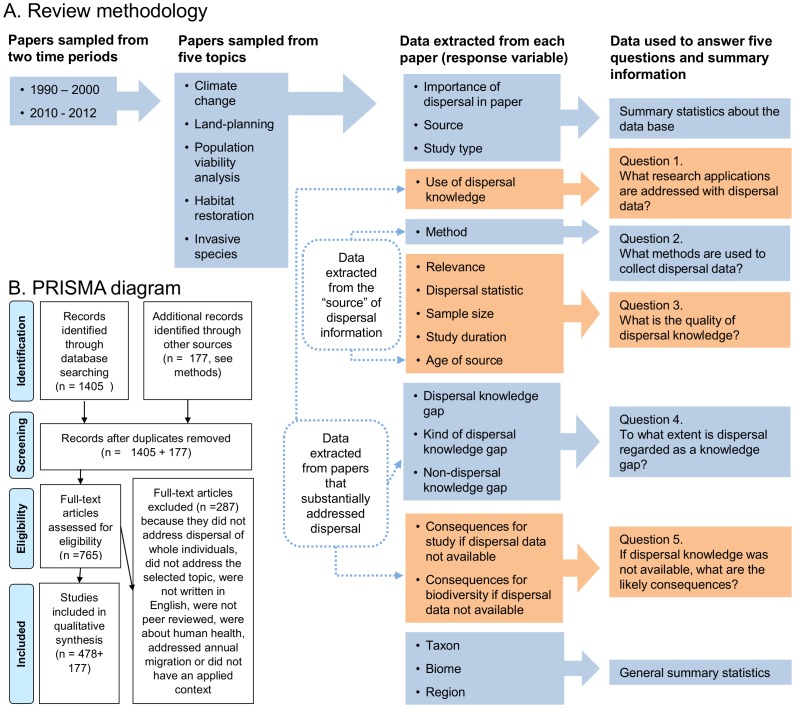
Overview of the review methodology. A. Sampling design, data collected and research questions. B. Flow chart based on PRISMA protocols [202] illustrating how papers were selected or discarded. Of the original 1405 papers, 765 were assessed for eligibility because papers were subsampled based on order of appearance in the literature (as detailed in methods).

To examine the trajectory of dispersal research in our five topics, we systematically reviewed dispersal-related literature. Using the ISI Web of Science in April 2012, we identified papers that included any one of five key words associated with dispersal (dispersal, connectivity, corridor, migration, colonisation) and keywords for each topic (Table 1). Web of Science subject areas were constrained to include environmental sciences and ecology, zoology, plant sciences, biodiversity conservation, marine and freshwater biology, entomology and forestry.We identified 1405 papers using our search terms (Figure 1). We note that using the ISI data-base in comparison to other literature data-bases (e.g. Scopus or Google Scholar) may have biased our selection of papers towards studies published in higher impact journals [85]. Given the high overlap of papers covered by the different data-bases, however, any bias is unlikely to alter our findings.

**Table 1:** Number of papers reviewed in each topic and time period.

Topic	Old	New	Topic search terms
Climate change	1990–1997 (115, 50)	2012 (160, 50)	climate change; climate-change
Invasive species	1990–1994 (151, 51)	2012 (124, 52)	invasive; exotic; alien
Land planning	1992–2000 (29, 18)	2010–2012 (72, 49)	landscape planning; landscape ecological planning; ecological planning; (conservation planning AND reserve)
Population simulation	1991–1999 (113, 50)	2010–2012 (130, 53)	Population viability analysis; PVA; simulation; population model; stochastic model; and one of: threatened; endangered; conservation concern; vulnerable; IUCN red list; extinction
Restoration Ecology	1990–1996 (134, 52)	2011–2012 (377, 53)	restoration

Time periods include Old (1990–2000) and New (2010–2012), with dates of each time period indicated (number of papers identified using search terms and number we reviewed in parenthesis). Search terms for topics are in addition to the search terms for dispersal (see text).

Our goal was to review approximately 50 papers in each of five topics and two time periods. A sample size of 50 was selected as a trade-off between what we could manage to review within a reasonable time-frame and the need for substantial sample sizes to distinguish trends. For the first time period, papers were sampled beginning with papers from 1990 (or the closest date after that when none were available from 1990) and sampling forwards until 50 papers were selected for each topic. For the second time period, we began sampling papers from the latest publication in April 2012 and sampling backwards in time until 50 suitable papers were selected for each topic. The time-periods therefore differed among topics, but we maintained a minimum of nine years between the early and late sample set (Table 1). To achieve this nine year gap, one topic with few papers (Land planning) was constrained to 2000 or earlier for the first time period, although 50 papers were not identified (Table 1).

While attempting to reach our target of 50 papers per topic and time period, 287 papers were rejected because they did not address dispersal of whole individuals, did not address the selected topic, were not written in English, were not peer reviewed, were about human health, were about annual migration, or did not have an applied context. The risk that bias might be introduced by special editions, repeated studies on the same species or by multiple publications from the same author were negligible (Table 1, Appendix S1). We reviewed 478 papers and examined an additional 177 source publications, which included 159 peer-reviewed papers, six theses, four long-term data bases and eight books. None of the source papers were also reviewed themselves so there are no papers that contribute directly to the data set more than once. Protocols are summarized in Figure 1 and Checklist S1.

### Reviewing Procedure

Nineteen people reviewed papers with each contributor reviewing up to three papers in each topic and time period. The even allocation of papers to reviewers ensured that no bias was introduced among topics and times, while mean differences among reviewers were accommodated in the analysis. Reviews were undertaken in two phases. First, each reviewer read one paper from each topic and time period, followed by a workshop to develop categories of responses for eighteen response variables (Fig. 1) that all reviewers could use consistently. In the second phase, first-phase reviews were up-dated using the newly defined categories, and an additional two papers were reviewed in each time period and topic by each person.

For each paper included in the review, we collected data addressing up to 18 response variables that were either related to answering our research questions or were summary statistics (Fig. 1, with details in online supporting information Table S1). To answer questions 3, 4 and 5, we collected data from papers that substantially addressed dispersal (defined as papers in which dispersal was included as an aim, used in analysis, or used in interpretation). Papers that did not substantially address dispersal (defined as papers in which dispersal was only briefly mentioned in the introduction) could not be used for these three questions. To answer questions 1 and 2, we collected data from the ‘source’ of dispersal information. In some cases, the source was the paper being reviewed (e.g. when the paper reported on dispersal findings), but in other cases the source was another piece of work (e.g. a reference to an earlier paper). In these cases, we located the additional source paper and used this to collect our data. Categories of responses and sample sizes for each category are provided in Table 2.

**Table 2:** Response variables used for addressing review questions (listed at the end of the introduction) and summary statistics.

Question	Response	N	Categories
Summary statistics	Importance of dispersal in paper	478	Aim/main-focus-of-paper (139); used-in-analysis (133); used-in-interpretation (206)
	Source	478	Current-paper (278); other-paper (159); book (8), thesis (6), long-term database (4), no source identified (23)
	Study type	478	Empirical (250); model including some empirical data (126); model or theory no empirical data (37); review (65)
Question 1	Use of dispersal knowledge	458	Different categories in each topic (Table 3)
Question 2	Method	423	Habitat occupancy (94); expert opinion (60); modelling (38); measure arrival from known sources (32); review (29); genetics (29); mark-recapture (28); theoretical (25); radio-tracking (18); seed traps (12); direct observation (11); compositional similarity among sites (7); inference based on traits (7); sediment cores (6); pollen or diaspore counts on animals (6); long term monitoring (5); aerial photographs (3); stable isotope analysis (3); estimated arrival dates based on organism growth rate and current size (2); GPS tracking (2); inference based on habitat quality (1); landholder questionnaires (1); pollen or diaspore counts on animals (1); satellite tracking (1); sediment cores (1); simulation of wind dispersal (1)
Question 3	Relevance of source paper	303	Same species, same environment (214); different species and/or different environment (89)
	Dispersal statistic	367	Inferred-dispersal-from-occupancy-data (148); dispersal-single-value (71); dispersal-distribution (66); inferred-dispersal-in-categories (38); genetics-inferred-dispersal (23); number/proportion-of-individuals-that-move (21)
	Sample size	478	Paper without a sample size (205); with a sample size (273) (papers with a sample size analysed as a continuous variable)
	Study duration	263	Median = 2 years, interquartile range = 1–4 years
	Age of source	163	Median = 7 years, interquartile range = 3.5–13 years (where source was other paper, book or thesis)
Question 4	Dispersal knowledge gap	467	None identified (252); dispersal identified as a knowledge gap (215)
	Kind of dispersal knowledge gap	214	Dispersal distance (52); behaviour (including timing, orientation, triggers, variation among individuals) (41); vegetation-specific dispersal (41); dispersal success (21); dispersal vectors (19); dispersal rate (17); methodological limitations (16); temporal variation in dispersal (2); source of colonists (2); dispersal undefined (2); home range size (1)
	Non-dispersal knowledge gap	466	None identified (212); one or more identified (254)
Question 5	Consequences for study if dispersal data not available	464	Conclusions-/-interpretation-from-study-weakened-/-unreliable (238); makes-no-difference (65); part-of-study-not-possible (42); entire-study-not-possible (112)
	Consequences for biodiversity if dispersal data not available	454	Cannot-predict-ecological-processes (196); cannot-determine-effectiveness-of-management-actions (128); cannot-predict-effects-of,-or-adaptation-to,-climate-change (55); cannot-model/predict-extinction-risk (36); none (39)
General summary statistics	Taxon	478	Plant (173); insect (52); mammal (46); bird (36); non-insect invertebrate (34); fish (32); ecosystem (18); vertebrates (119); none (10); invertebrates (86); lichen (5); fungi (10); amphibian (7); reptile (4).
	Biome	441	Freshwater (65); marine (44); terrestrial (348) (some studies include >1 biome)
	Region	424	Global (36); Africa (26); Europe (101); North America (163); South America (20); Australasia and Pacific (46); Asia (Pakistan through to Japan and Indonesia) (20)

N  =  number of reviewed papers used in the analysis. N varies among response variables because some responses refer to a subset of papers, and some papers could not be assessed for particular responses. The number of reviewed papers in each category is given in parentheses.

### Analysis

We converted each level of categorical response into separate binomial responses, with a separate variable created for levels with more than 20 positive records. For example, we recognized three levels within the biome response (terrestrial, freshwater, marine), and we generated three separate binomial variables, with the first being terrestrial studies versus not terrestrial studies. This approach inevitably meant that a strong pattern in one response level was likely to be mirrored by one or more other response levels. We analysed these with binomial generalized linear mixed models [86], fitting topic, age and their interaction as fixed effects, and reviewer as a random effect.

Study duration in years, and sample size were analysed using a Poisson generalized linear mixed model, with the same fixed and random predictors as the binomial models. Sample size was calculated in a method-specific way and study duration was dependent on method, so we fitted method as an additional random effect to those responses. To account for over-dispersion of the Poisson models, we also fitted an observation-level random effect [87]. Analyses were performed using the lme4 library [88] and parameter estimates were obtained with AICcmodavg 1.24 [89] with R 2.15.2 [90].

We focus on results where P<0.05, but also discuss results where 0.05< P<0.1 and the estimated confidence limits for levels do not substantially overlap. This approach minimises the risk that any trends in the data are overlooked and we regard the risk that some results may be false positives as acceptable. P values indicated on figures are for the significant (P<0.05) or near-significant (0.05<P<0.1) factor that has been plotted. Papers were given equal weighting in the analyses.

## Results

We first present results for three summary statistics that provide insight into the nature of the database, followed by answers to our five research questions and finally summary statistics for taxon, biome and region. Test statistics for all analyses are provided in Table S2 and a summary of effects is provided in Table S3.

### Summary statistics: the nature of the database

#### Importance of dispersal in paper

Twenty nine percent of the studies that we reviewed had dispersal as their main focus or aims while 28% used dispersal in the analysis. However, the largest proportion (43%) of papers in the review were not specifically conducted to learn about dispersal, rather, dispersal information was used in interpreting related information, with dispersal mentioned in the discussion (Table 2).

**Figure 2:**
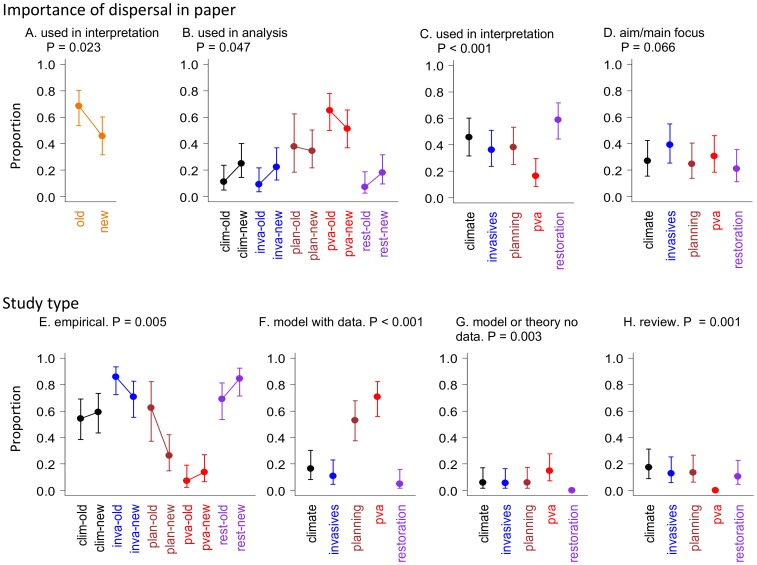
Importance of dispersal in papers and study type. For factors that varied with topic, time or their interaction: the importance of dispersal in the reviewed papers (A – C), and; the study type (D – G). Responses indicate the proportion of reviewed papers with, for example, dispersal as the aim/main focus (A). Error bars indicate 95% confidence limits. Categories for age by topic interactions are indicated with abbreviated topic names (clim  =  climate change, inva  =  invasive species, plan  =  land planning, pva  =  PVA, rest  =  restoration). Old refers to papers from the first time period (1990s), while “new” indicates papers from (2010-12).

The proportion of papers using dispersal for interpretation has declined over the past ten years (Fig. 2 A), with evidence of a shift towards using dispersal in analysis in three of the five topics (Fig. 2 B). A high proportion of restoration studies used dispersal information in their discussions only (Fig. 2 C). There were only weak differences among topics in the proportion of studies for which gathering dispersal knowledge was a main aim (Fig. 2 D).

#### Source of dispersal knowledge

The source of dispersal knowledge for most papers (58%) was the paper itself (Table 2) although 33% of reviewed papers cited other research as their source of knowledge. These proportions did not vary significantly with age or topic.

#### Study Type

Approximately half (53%) of the studies reviewed were empirical, one quarter (26%) were modelling studies with some empirical data, 8% were modelling or theoretical studies without data, and 14% were reviews. For every 6.4 empirical, modelling or theoretical papers that examine dispersal, there was one review.

The proportion of empirical studies in land planning has declined over the past ten years (from > 0.6 to<0.3, Fig. 2 E). The majority of land planning and PVA papers were modelling studies (Fig. 2 F–G). Restoration had very few modelling studies, and none that were entirely theoretical (Fig. 2 F–G). PVA had only two reviews, and meaningful confidence limits could not be estimated (Fig. 2 H).

### Research questions

#### 1. What research applications are addressed with dispersal data?

For each of the five topics, we identified a small number of broad research applications. The research applications were addressed in approximately the same proportions across time periods within topic, and each time period addressed a similar number of research applications (Table 3). Although sample sizes were small and our Fischer's exact tests were all non-significant, changes in applications over time suggested by our data included an increase in invasive species papers that investigate specific control methods (from 1 to 7). In the climate change topic, there was an increase in the number of papers addressing adaptive potential (from 2 to 9, Table 3).

**Table 3:** Research applications addressed by dispersal-related papers*, the number of papers addressing each in the Old and New time periods.

Main way that dispersal knowledge was used or the problem to which it was applied	Topic	Old	New	P
Evaluate adaptive potential	climate	2	9	0.25
Assess colonisation potential	climate	9	8	
Describe current patterns of occurrence	climate	8	7	
Identify refugia and colonisation routes	climate	0	1	
Predict impact or extinction risk associated with climate change	climate	11	12	
Predict or measure range shifts in distribution	climate	17	12	
Describe the impact of invasive species	invasives	6	5	0.20
Assess the influence of environmental factors on invasive species/invasion	invasives	4	3	
Identify the invasion mechanism	invasives	12	7	
Describe the present distribution of invasive species	invasives	21	16	
Investigate specific control methods, including biocontrol	invasives	1	7	
Predict the future spread or distribution of invasive species	invasives	6	9	
Determine the best spatial arrangement of patches/reserves/habitat in the landscape: Global scale	land planning	1	7	0.94
Determine the best spatial arrangement of patches/reserves/habitat in the landscape: National scale	land planning	2	6	
Determine the best spatial arrangement of patches/reserves/habitat in the landscape: Local scale	land planning	12	27	
Determine where management actions should be carried out: National scale	land planning	0	1	
Determine where management actions should be carried out: Local scale	land planning	1	3	
Predict consequences of future land-use change (urban development etc.): Local scale	land planning	2	4	
Assess demography and population change	PVA	34	31	0.29
Assess effect of climate change	PVA	0	5	
Evaluate species interactions	PVA	4	2	
Predict effects of landscape variation	PVA	9	9	
Predict sustainable harvest	PVA	2	2	
Plan restoration of degraded areas	PVA	0	1	
Simulate population migration	PVA	1	1	
Design restoration, taking into account dispersal mechanism or rate	restoration	19	19	1.00
Determine whether restored habitat will be naturally colonised, or if translocation needed	restoration	19	18	
Incorporate connectivity into restoration	restoration	12	11	

P  =  P value of Fisher's Exact test. There were also no significant differences when questions addressed by less than 10 papers were excluded from the test. Note there were only 18 completed reviews among old land planning papers so old and new numbers are not directly comparable.

*Our categories of research were developed during workshops with the authors and were based on one-third of the papers that we reviewed. The research questions that we identified therefore arise from the papers. However, we acknowledge that there may be other ways that the broad categories of research might be defined, and this may reveal different insights into the nature of changing research questions over time.

#### 2. Which methods are used to collect dispersal data?

The most frequent methods used to gather dispersal data were habitat occupancy (22%), expert opinion (14%), and modelling (9%). Direct tracking methods together accounted for 14% of studies, including: mark-recapture (7%), radio-tracking (4%), direct observation (3%), GPS (0.5%), and satellite tracking (0.2%). For a full list of methods by topics and age, see Table S4.

**Figure 3:**
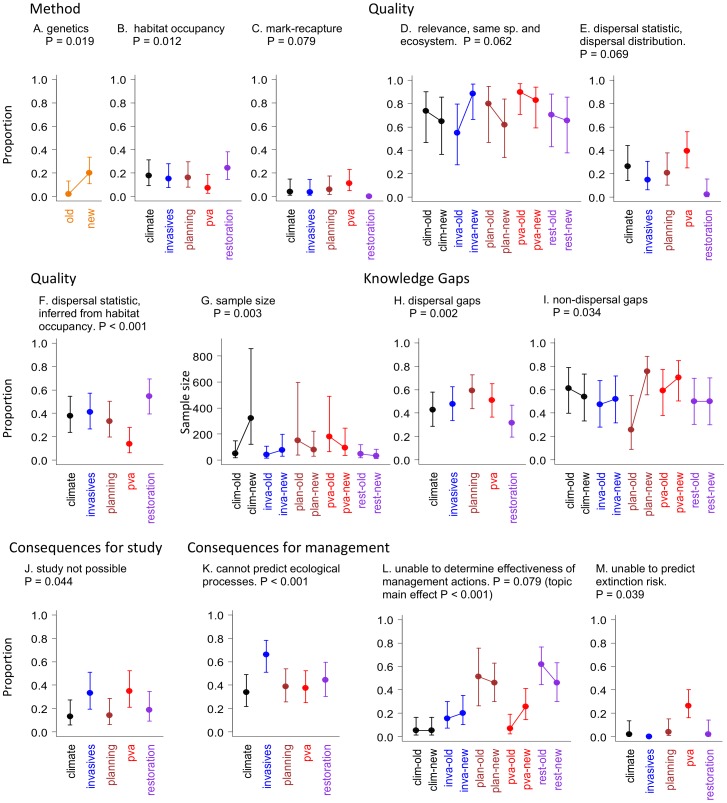
Effects related to the five research questions. For factors that varied with topic, time or their interaction: the method used to obtain dispersal data (A-C); the quality of the dispersal data, including relevance (D); dispersal statistic (E, F); and sample size (G); dispersal knowledge gaps (H); non-dispersal knowledge gaps (I); consequences for the study (J), and; consequences for management (K-M). Error bars indicate 95% confidence limits.

The only temporal change in the methods used to collect dispersal data was an increase in the application of genetics across all topics over the past 10 years (Fig. 3 A). Habitat occupancy methods were applied less often in PVA than in restoration (Fig. 3 B). Few restoration studies used mark-recapture methods and confidence limits could not be estimated (Fig. 3 C). Other methods were applied at similar rates across topics and across times.

#### 3. What is the quality of dispersal knowledge?

Relevance: Papers of high relevance (i.e.: same species from the same ecosystem) occurred more often in recent invasive species literature than in older literature (Fig. 3 D). Other topics used papers with similar relevance in each time period.

Dispersal Statistic: There has been no change in the rate at which different dispersal statistics are used. Inferring dispersal from occupancy data was most common (40%), while 19% of papers used a single value of dispersal, such as maximum dispersal distance (see Table S1, Table 2). A similar proportion of studies used a dispersal distribution (18%) but very few (6%) used genetic statistics. Dispersal distributions tended to be used more often in PVA and very rarely in restoration studies (Fig. 3 E). Restoration papers frequently inferred dispersal from habitat occupancy (Fig. 3 F).

Sample Size: Sample size has increased in recent climate change research compared with older climate change literature and is larger than in most other topics in any time period (Fig. 3 G).

Study Duration: Study duration (median  =  2 years, interquartile range  =  1–4) did not vary significantly with topic, time period or their interaction (Table 2).

Age of Source: The age of the source material (median  =  7 years, interquartile range  =  3–13) did not vary with age, topic or their interaction.

#### 4. To what extent is dispersal regarded as a knowledge gap?

Limited dispersal knowledge was identified as an impediment or research gap in 46% of studies. Fewer papers cited dispersal as a research gap in restoration ecology compared with the highest rates in land planning (Fig. 3 H). The most commonly cited aspects of dispersal in need of further research were dispersal distance (24%), dispersal behaviour (variation among individuals, timing, orientation, triggers) (19%) and vegetation-specific dispersal (19%) (Table 2).

The majority of studies (55%) cited factors other than dispersal as impediments to research progress or knowledge gaps. The number of non-dispersal limitations was lowest in old land planning research, but this increased to be among the highest mean values in recent papers (Fig. 3 I).

#### 5. What would be the likely consequences for research and conservation if dispersal knowledge was not available?

Conclusions would be weakened in 51% of studies in the absence of dispersal data, and almost one quarter of studies (24%) would not have been possible (most of these directly examined dispersal). For an additional 9% of papers, part of the study would not have been possible. The lack of dispersal data would have made no difference to 14% of studies. Among the 24% of studies that would not have been possible without dispersal data, a higher proportion occurred in the invasive species and PVA topics (Fig. 3 J).

If dispersal knowledge had not been available, the consequences for making conservation decisions could be allocated to five broad categories. For a small number of papers (8%) there would be no consequence of lack of dispersal knowledge. Lack of dispersal data would limit knowledge of ecological processes in 43% of papers, but this problem was most common in the invasive species topic (Fig. 3 K). The effectiveness of alternative management actions would remain unknown in 28% of papers. However, this problem was twice as common among land planning and restoration papers, and there was weak evidence that this has increased recently in the PVA topic (Fig. 3 L). Predicting extinction risk would not be possible in 8% of papers, which was most commonly a problem in PVA studies (Fig. 3 M).

### Summary Statistics

#### Taxon

Over one third of studies in our review focussed on plants (36%), one quarter of studies focussed on vertebrates (25%) and fewer on invertebrates (18%). The 26% of studies on vertebrates were composed of 9% on mammals, 8% on birds and 7% on fish. Reptiles and amphibians accounted for 1.4% and 0.8% respectively. Most of the invertebrate studies were on insects (11%).

**Figure 4:**
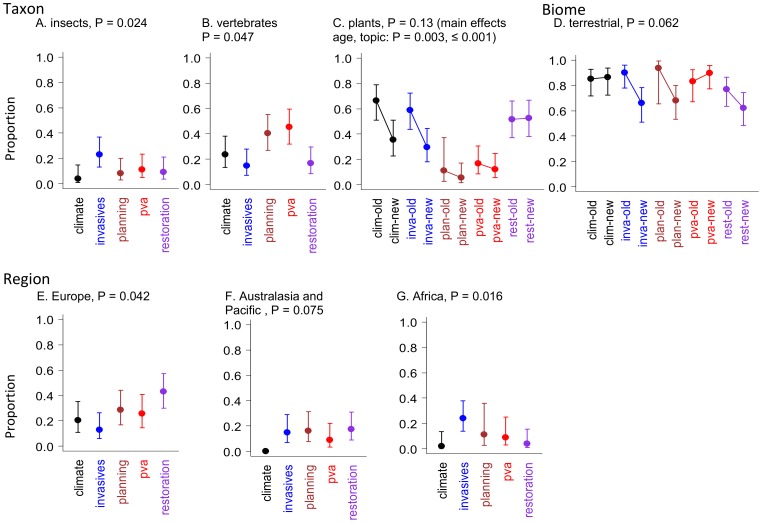
Effects related to Taxon, Biome and Region. For factors that varied with topic, time or their interaction: the frequency of papers that studied particular taxa (A-C); the biome they focussed on (D), and; the region of the world from which they were from (E-G). Error bars indicate 95% confidence limits.

Insects were most frequently studied in the invasive species topic (Fig. 4 A), and this pattern was also evident in the broader group, invertebrates (P = 0.011, result not shown). Vertebrates were more frequently the focus of PVA and land planning studies compared with the invasive species and restoration topics (Fig. 4 B). Conversely, plants were least commonly studied in land planning and PVA papers, but were the focus of over half of the restoration studies (P<0.001). The proportion of studies examining plants has declined in the past ten years (P = 0.001), although the interaction of age and topic (P = 0.13) indicates that substantial declines only occurred in the climate change and invasive species topics (Fig. 4 C).

#### Biome

Over three quarters of papers reviewed were from terrestrial ecosystems (79%), with 15% from freshwater and 10% from marine ecosystems (some studies included more than one biome). The proportion of studies examining terrestrial ecosystems has declined in the invasive species, land planning and restoration topics (Fig. 4 D).

#### Region

Most studies were undertaken in North America (38%) and Europe (24%) with 11% in Australasia and the Pacific, 6% in Africa, and 5% in South America (Table 2). Studies from Europe most commonly addressed dispersal in a restoration context, and least often in an invasive species context (Fig. 4 E). Studies from Australasia and the Pacific (Fig. 4 F) and Africa (Fig. 4 G) had only a small proportion of papers that examined dispersal and climate change.

## Discussion

Dispersal knowledge is essential for conservation management, but is dispersal knowledge growing at a pace that can meet dispersal demand? There was some evidence that dispersal has increased in importance, with a decline in the proportion of papers using dispersal for interpretation only (Fig. 2 A). However, more generally, our results showed that dispersal data are typically of low quality, and that there has been limited improvement in data quality or growth in application over time. We next discuss our findings regarding the five research questions and then provide a ‘report card’ for each of the five topics. We finish by suggesting approaches that are needed to improve collection and application of dispersal data.

### 1. What research applications are addressed with dispersal data?

We did not detect significant growth of new applications in any of the topics or a major change of emphasis, at least at the coarse scale of our categories of questions. When research applications across fifty papers are consolidated into a handful of broad categories, it is inevitable that the applications must become general and lose some ability to distinguish progress in the field. Nevertheless, absence of significant changes over time indicates there has not been a major shift in any of these fields for the past 10 years. Even though new problems are emerging in conservation biology [34], the same broad issues that we knew about in the 1990s remain issues of concern today (Table 3). This may in large part reflect under-resourcing and limited implementation of conservation action [91]–[94]. Threats to biodiversity of 10–20 years ago therefore remain significant threats and so continue to attract research attention [93], [94].

The number of papers addressing some applications (Table 3) changed substantially between time periods, so these may be important areas of growth. In the climate change topic, papers examining adaptive potential increased from two to nine (Table 3). A recent climate change review [95] urged more attention in the area of adaptation in relation to range shifts. Our review raises the possibility that this knowledge gap is beginning to be addressed. In the invasive species topic, the number of papers investigating specific control methods, including biocontrol, increased from one to seven (Table 3). The increased number of papers in this category may suggest a change in emphasis from documenting invasive species impacts, to finding new methods for control, indicative of progress in this topic.

### 2. Which methods are used to collect dispersal data?

The three most commonly applied methods for studying dispersal were habitat occupancy (22% of studies), expert opinion (14%) and modelling (9%). Few papers directly measured dispersal, and the majority of papers that discussed dispersal were based on indirect inferences. This appears not to have changed over the past 10 years. The general lack of change across time suggests that, apart from genetics, there is stagnation in the way ecologists measure dispersal. While it is possible to select specific studies that demonstrate exciting new applications of technology for measuring dispersal [96], when taking a sample of dispersal related papers from the broader literature, those new approaches are yet to have an impact.

We next discuss the increasing use of genetics, and then consider the quality of dispersal data that arises from the three most commonly applied methods: occupancy, expert opinion and modelling.

#### Genetics

The application of genetic analysis to dispersal studies is increasing [97], reflecting the strengths of new genetic approaches. Genetic markers have become increasingly available, from microsatellites in the 1990s to new DNA sequencing methods that enable rapid genome-wide marker development [98], [99]. In addition, new statistical approaches have been developed for inferring dispersal from genetic information [32], [100]–[102]. Genetic methods can often be applied in situations where more direct approaches cannot [103]. This has created opportunities for studying dispersal questions that would not otherwise be tractable, such as reconstructing colonisation or invasion patterns [104], or for studying dispersal in taxa that are difficult or impossible to track, such as plants [105], [106].

Genetic approaches have a range of limitations that mean they are not the panacea of dispersal research. Classic population genetics theory proposes that the number of migrants per generation can be estimated from genetic differentiation, such as F_ST_ [107]. However, the underlying assumptions about population structure are rarely met, and processes other than contemporary dispersal can drive population differentiation, particularly as the scale of sampling rises [20], [102], [108], [109]. Increasingly, spatial patterns of genetic distances between individuals or populations are used to test hypotheses about relative dispersal rates [100], [110]. These can contribute to our knowledge of how landscape heterogeneity affects population connectivity, but do not directly quantify dispersal. Indirect methods based on genetic divergence integrate dispersal over many generations, meaning that genetic estimates of dispersal exceed the distance that any one individual disperses [20]. Further, because genetic structure is a consequence of multiple generations of dispersal, there can be lag effects in landscapes subject to recent change, where current genetic structure reflects historic dispersal patterns [111]. Landguth et al. [112] suggested lag effects would be longer after fragmentation than after removal of dispersal barriers. Further, different genetic metrics have differing time lags of sensitivity to changed dispersal: methods based on spatial patterns of multi-locus genotypes respond more rapidly (as little as 1-5 generations) than methods based on allele frequency differentiation, such as F_ST_ [112].

In contrast with indirect genetic methods, genetic assignment tests [101], parentage analyses [113] and genetic tagging [114] can be used to obtain some of the same individual-level dispersal data that long-term radio-tracking or mark-recapture studies can provide [115]. However, there are constraints on when these techniques can be applied. Assignment tests require substantial sample sizes and levels of genetic differentiation, and comprehensive sampling of potential parents is needed to infer dispersal from parent-offspring locations [103]. Parent-offspring analysis also faces the challenge that offspring may not have finished dispersing when sampled, and that parents may disperse after giving birth [20]. Genetic methods have substantially enhanced our toolbox for studying dispersal, and the kinds of dispersal processes that can be examined. However, it is important to understand the conditions under which genetic analyses are applicable, the links between different types of genetic data and individual dispersal rates, and thus what kinds of dispersal information we can and cannot obtain from genetic analyses. Genetic analyses may best be applied in combination with direct approaches because they can provide complementary information [12], [103], [116], [117].

#### Occupancy

The most common method used to infer dispersal was habitat occupancy, probably because it relies on survey data that are easy to collect relative to other methods. The quality of occupancy data used to infer dispersal varied substantially. Six studies (out of 94) used occupancy modelling to provide powerful insights into dispersal. For example, to estimate metapopulation parameters for two wetland bird species in California USA, Risk et al. [118] applied stochastic patch occupancy models (SPOMs) to data from 228 sites collected over six years. Average dispersal distance was estimated to be approximately 10 km for the black rail *Laterallus jamaicensis*, but over 1000 km for the Virginia rail *Rallus limicola* [118].

Other approaches were applied more often than stochastic patch occupancy models in dispersal-related research, and these typically provided less specific information about dispersal. For example, statistical models were used to relate occupancy data to potential dispersal corridors in the surrounding landscape [119]. This approach was often used in land planning, where occupancy data were used to model the relationship between measures of structural connectivity and species diversity [120], [121]. In restoration, presence at a restored site can be related to occurrence of possible sources in the landscape [59]. An even simpler approach was to address whether colonization occurs after habitat creation or enhancement, by examining the species present in restored sites [122], with inference constrained to whether dispersal is limited or not.

While there is a range of approaches for extracting dispersal information from habitat occupancy data, this method imposes substantial constraints. Most generally, individuals are not considered and therefore there is no information about dispersal mechanisms. For example, in invasive species research, behavioural knowledge may be the key to understanding why some species are successful invaders and others are not [123], but this kind of information is not considered in occupancy approaches. When mechanisms are not considered, the correlative inferences about dispersal from occupancy in one landscape may not transfer to other landscapes or times [19]. Stochastic patch occupancy models have some specific limitations, including a requirement for large data sets and an equilibrium between colonisation and extinction [124], [125].

A combination of the limitations associated with occupancy data may have contributed to the findings of Poos and Jackson [63], who developed stochastic patch occupancy models for the freshwater fish redside dace *Clinostomus elongatus* in Canada. The estimated time to extinction was up to orders of magnitude shorter when direct estimates of dispersal from mark-recapture data were used, compared with using a dispersal kernel based on patch-occupancy [63]. This discrepancy raises a major challenge to using occupancy (or mark-recapture) data alone to parameterise metapopulation models and highlights the urgent need for research to understand the basis of these conflicting results. Long-term field datasets and direct estimates of dispersal will be particularly important to improve the quality of dispersal data. Long term data-sets could help identify some of the limitations of occupancy data, such as when there is spatially correlated extinction [126] and whether the system is in a colonisation-extinction equilibrium.

#### Expert opinion

Expert opinion was the second most common “method” for acquiring dispersal information. Dispersal clearly remains a major knowledge gap because data would have been used in preference to expert opinion if it had been available. Although often applied, expert opinion is rarely validated [75]. Studies assessing the value of expert opinion suggest that expert opinion does not help to improve predictions. For example, bird distribution models were no better at predicting distributions when expert opinion was incorporated [127]. Zeller et al. [97] were critical of the application of expert opinion for assessing landscape resistance, pointing out that it usually leads to poor parameter estimates. In a meta-analysis of butterfly dispersal, Stevens et al. [128] found that expert opinion reflected the tendency of species to migrate and not the likelihood of dispersal between populations. On the other hand, the use of expert opinion, if properly elicited, can provide valuable priors for Bayesian analyses [129]. Similarly, expert opinion may be a useful starting point for estimating landscape resistance, when followed up by subsequent optimization approaches using additional genetic data [130]. There seems to be little support justifying the use of expert opinion beyond its application as a starting point for further analyses. Continuing dependence on expert opinion for dispersal knowledge indicates that collection and application of dispersal data needs to be improved.

#### Simulation Modelling

Dispersal information was the output of (and often the input to) a simulation model in 9% of studies, the third most common method. In some cases, modelling was used to overcome difficulties associated with direct tracking, and where there was a mechanistic understanding of how species spread. Hence, dispersal of fungal spores, terrestrial seeds and marine larvae was estimated by modelling air and water flows [131]–[133]. In contrast, most papers that used modelling as a method were based on either vegetation surrogates [134], theoretical dispersal distributions [135], or arbitrary parameters [136], [137]. Some of these studies are “thought experiments” and actual dispersal knowledge will be needed to discover which of the possible mechanisms that were explored in theory really occur in nature. Studies using surrogates and theoretical dispersal distributions require testing to understand the extent to which they adequately represent systems in nature [75]. Theoretical dispersal models, such as random walks, omit critical aspects of behaviour that can influence dispersal and therefore may not predict actual dispersal very well [138]. In addition to empirical tests of model assumptions, global uncertainty and sensitivity analyses will be valuable for discovering the extent to which dispersal knowledge is needed to successfully model populations [139].

### 3. What is the quality of dispersal knowledge?

Our indices of the quality of dispersal data suggested that quality is often low. Among the papers we surveyed, dispersal distributions represented relatively high quality dispersal data, but these were applied in 18% of cases. Together with single values for dispersal, dispersal statistics consisted of actual distance estimates in just over one third of papers. Two thirds of papers used indirect measures, mostly inferences based on occupancy, with the limitations discussed previously. One exception to the wide use of low quality dispersal data was our index of the relevance of dispersal knowledge; most papers used dispersal data from the same species and ecosystem to which the information was applied.

We expected that the age of source papers might indicate a change in the quality of dispersal data. If younger source papers were used among the 2010–2012 papers compared with papers from 1990–2000, it would imply an improvement in the quality of data that are available. If younger papers are used, these likely offer the most valuable insights. In contrast, an increasing age of source papers would imply that all of the good work was done in a specific historic period, with no recent improvement. The observed absence of change implies that neither of those extremes has occurred; the quality of dispersal data in recent papers is about the same as they were in the 1990s. More detailed analysis than we were able to do is needed to examine the underlying causes of the lack of trends in the field, but may require new data on social constraints, such as funding limitation [140] and transfer of knowledge and research culture from academics to students [141], [142].

### 4. To what extent is dispersal regarded as a knowledge gap?

Almost half of the papers we reviewed identified knowledge gaps related to dispersal. The main dispersal knowledge gaps were those related to dispersal distance, dispersal behaviour, and spatial variation in dispersal in different vegetation types. Dispersal is therefore recognised as a complex phenomenon in a substantial proportion of papers, and this has not changed over the past ten years. Previous reviews have emphasised that dispersal is complex because it is conditional on a range of external environmental states and internal physiological and behavioural states [16], [36], [143], [144]. Recommendations to further recognize this fact [16] remain warranted. However our results suggest recognising that dispersal is complex may not be the main limitation. The main limitation relates to how to gather information about the diverse range of factors that influence dispersal, a point we return to later.

Although dispersal is an important knowledge gap in conservation biology, it is not the only knowledge gap. Over half of the papers that we reviewed identified knowledge gaps unrelated to dispersal. From our assessment of papers, dispersal was as important as other knowledge gaps. For example, Chapple et al. [123] recognised a range of knowledge gaps regarding traits that influence invasiveness of exotic species, including dispersal, but also behavioural traits such as parental care. In describing a land planning simulation model, Gurrutxaga et al. [145] admit that they did not use empirical data to establish landscape resistance to dispersal, and they did not take into account all dispersal pathways. However, detailed habitat mapping, including distinguishing primary forest from plantations, also remained an important knowledge gap and this probably was as limiting as gaps related to dispersal.

### 5. What would be the likely consequences for research and conservation if dispersal knowledge was not available?

We predict there will usually be serious consequences when dispersal knowledge is not available. We identified few papers where there would be no consequences for biodiversity and management if dispersal knowledge was not available. If dispersal data were lacking, extinction risk could not be estimated (arising from approximately one quarter of PVA studies), the effectiveness of management actions could not be predicted (inferred in 28% of studies), and understanding of ecological processes was foregone in 43% of cases. When dispersal data are not available, knowledge that is important in a conservation context is not gained, and it becomes difficult to distinguish among management options. For example, lack of information about mollusc dispersal between streams meant that the likelihood of colonisation of restored streams, and rates of movement to track shifting climatic niches were unknown [146]. The consequence of limited dispersal knowledge is that money spent on conservation may be at risk of being wasted, and conservation outcomes could be compromised.

### Taxonomic bias

Taxonomic bias was generally strong and widespread in our data set. Taxonomic bias has been well documented in the conservation literature for over ten years [39], and our study confirms that this has not changed (also see [147]–[149]). Bias towards mammals and birds is just as strong for dispersal-related research as it is more broadly across conservation [19], [39], [150], [151]. Despite being a well-established effect with substantial consequences for biodiversity conservation [152], [153], the scientific community has not moved to redress this bias. This inertia may have a number of contributing factors, including methodological limitations [154], publication bias [147] and societal preferences [148]. Stemming from societal preferences, ongoing taxonomic bias is contributed to by funding constraints [140], and a focus on charismatic species [155].

### Topic report cards

#### Climate change

Of the five topics we examined, climate change has the largest literature. A search of ISI Web of Knowledge using our key words (Table 1) and constraining the search to environmental sciences and ecology in 2012, revealed that the climate change literature is around one third bigger than our PVA topic, six times bigger than restoration, an order of magnitude bigger than land planning and two orders of magnitude bigger than invasive species. Not only is climate change the largest field, it also seems to be progressing in terms of quality and application of dispersal data. Climate change papers had the largest and increasing sample sizes and more papers use dispersal in analysis than they did 10–20 years ago. Climate change is a major threat to biodiversity conservation [51], [52], so it is good news that this field is showing some signs of improving its collection and use of dispersal data. However, climate change studies that address dispersal were substantially under-represented in Australasia and the Pacific and Africa. Climate change and connectivity are certainly important issues in those regions [23], [156], [157], and they remain clear knowledge gaps.

#### Restoration

In contrast with climate change, there appears to be substantial room for improved use of dispersal knowledge in restoration research. Generally low quality dispersal data were used, with very high dependence on occupancy studies and low application of mark-recapture research and dispersal distributions. There also were very few modelling studies which suggests that the restoration topic has not adequately grappled with the difficult large-scale, long-term processes that modelling papers often address. Restoration of ecosystems is, in most cases, a large-scale and long-term process [158] and would benefit from more consideration of landscape-wide and long-term phenomena [159]. Further, the ongoing debate about whether the amount of habitat is more important than habitat configuration [29], [30], [160] implies much remains to be learnt about how the spatial configuration of restoration influences recolonisation. Despite these knowledge gaps, restoration papers had the lowest rate of reporting dispersal as a knowledge gap (Fig. 3 H) and retain a strong focus on restoration of individual sites [159]. We suggest that the field of restoration ecology is yet to properly address the landscape context of restored sites, and as the field develops it may come to acknowledge the need for dispersal data more often.

Of the topics we considered, restoration was the most popular in Europe, a pattern not observed in other regions. The reasons for this are not clear, but could be because restoration in Europe has a focus on grassland communities. Approximately 40% of restoration studies from Europe focussed on grassland restoration which is a relatively simple community to manipulate, such as by mowing or seed addition [161], [162]. In contrast, less than 10% of restoration studies from North America focussed on grassland restoration.

#### PVA

Population viability analyses (PVA) provide something of a litmus test for the availability and efficacy of dispersal data because they require high quality data to forecast the fate of populations. Population simulation studies made relatively low use of occupancy data and had the highest use of dispersal distributions as the dispersal statistic. Use of mark-recapture as the method was among the highest of any topic. These observations suggest that occupancy data (the most commonly applied method) does not provide an adequately quantitative assessment of dispersal to use in simulation models. The implication is that much of the occupancy data currently used in the literature are of relatively low quality.

In a review of the PVA topic, Naujokaitis-Lewis et al. [66] found that dispersal is a key variable influencing modelling outcomes. But how much dispersal knowledge is needed? An important step to progress the application of dispersal in PVA is to test the predictive ability of simulation models that include different levels of dispersal realism. For example, models that consider only a single value of dispersal and some estimate of error [163] could be compared with models that incorporate many aspects of dispersal including empirically measured dispersal kernels [164], or models where movement depends on environmental conditions or the biological state of individuals [165].

If dispersal data were not available, the consequence implied by an increasing number of PVA papers is that the effectiveness of management actions will remain unknown. Papers that have highlighted the poor predictive value of PVA have nevertheless argued that PVA may still be able to effectively rank management alternatives [166]–[168]. The increasing number of PVA papers for which effectiveness of management would remain unknown if dispersal data were not available may imply that population simulation studies are becoming more explicit in attempts to distinguish among management alternatives. This is an encouraging trend that will help to make simulation modelling studies more relevant to conservation managers and policy makers.

The relatively small number of reviews in PVA is noteworthy. Disregarding topics, the overall rate at which review papers were selected in our literature search was very high; one review for every 6.4 papers that discusses dispersal. However, there was little redundancy among the reviews because most were narrow in scope [52], [71], [169]–[172]. The high rate of reviews among the papers we examined may arise because dispersal intersects a broad range of topics, and so appears in a diverse range of review papers. In contrast, the small number of reviews in the PVA topic arises because this “topic” is based on methodology, rather than the broader fields of research that link the papers in our other topics. Only specialised reviews focus on the PVA method [66], [173].

#### Land planning

Land planning papers frequently reported that dispersal was a knowledge gap (Fig. 3 H). Despite recognising that dispersal information is inadequate, the proportion of empirical studies is declining (Fig. 2 E). The gap between demand for dispersal information in land planning and the rate of acquisition of that knowledge is therefore increasing. The consequence is that, in the future, an even larger proportion of papers will not be able to discern how effective alternative management strategies may be, undermining effective conservation planning. Reviews of land planning research emphasise that connectivity is poorly understood, but some take the pragmatic approach that when ‘real information’ is not available, a surrogate is an adequate alternative [174]–[176]. However, surrogates may not be good proxies for dispersal [177]. Perhaps because surrogates are widely accepted as reasonable in this sub-discipline, there has not been a push to close dispersal knowledge gaps, even though the lack of dispersal knowledge is well recognised. The topic of land planning would likely benefit from placing more emphasis on collecting high quality dispersal data, incorporating those data into analyses and testing the utility of connectivity surrogates and expert opinion [72], [75]. Baguette et al. [73] provided a five step approach to improving connectivity measures in land planning simulations, including measuring dispersal in a range of umbrella species using genetics. We support this approach, although given the limitations of genetic methods, additional methods may be needed. Further, given the limitations of umbrella species [178]–[180], the benefits for other species of conserving a particular umbrella species would need to be demonstrated.

#### Invasive species

Invasive species studies use dispersal data with high relevance (from the same species and ecosystem) more often than they did 10–20 years ago. This is a positive sign that the quality of dispersal data in the field is improving. There was a decline of plants and terrestrial studies (and hence an increase in less common groups) suggesting that some of the taxonomic and biome biases present across all topics may be reducing in studies of invasive species. Pyšek et al. [153] implied bias was low in invasive species research because it includes research across the taxonomic spectrum.

Invasive species research was poorly represented among European studies. This perhaps partly stems from the difficulty of distinguishing invasive species in Europe, where glaciation and recent range changes have been widespread, and new species can be regarded as desirable [181]. In contrast, continents and islands with a shorter history of modern human occupation have suffered more severely from inter-continental invasive species, to the point that such invasions are a predominant cause of faunal collapses [23].

#### Improving dispersal knowledge and its application

For many organisms, dispersal knowledge is either not available or is of poor quality, limiting the effectiveness of biodiversity management. To improve the situation, we suggest there are three areas where further development is needed; we need more data of higher quality, we need to understand the extent to which dispersal data collected using different methods are congruous, and we need to know which data are required to distinguish among management options.

First, better dispersal data are needed. In discussing the movement ecology approach [144], Holyoak et al. [19] argued that more ambitious research projects are needed. Multidisciplinary projects that examine dispersal physiology, behaviour and external constraints in addition to measuring distances moved are needed to progress the field [19], [146]. Improving the quality of dispersal knowledge will involve developing more sophisticated analytical approaches [32], [143], [182]–[184], and applying new technology to expand the scope for data collection [144], including new methods for tagging and tracking small animals [96], [185]–[188], discovering new applications for acoustic surveys [189], and improved genetic methods [20], [98], [99]. While genetic methods for measuring dispersal are often advocated [73], it is important to remain aware of the strengths and limitations of different approaches.

The second area for improvement is to understand the extent to which methods are interchangeable or complementary. Indirect genetic methods sometimes provide dispersal knowledge that is congruent with direct ecological methods [190], [191], but not always, and this remains an area for further research [20]. In other circumstances ecological and genetic methods for measuring dispersal are complementary [103], [116], [117]. For example, genetic assignment tests can measure seed dispersal, but cannot provide information about dispersal behaviour of the animal vector, so may have limited predictive value [182].

To make progress, major research projects are needed that examine dispersal in a range of species and simultaneously using a range of methods. Results from a movement ecology approach, for example, could be used to test inferences about dispersal arising from occupancy data. Ambitious movement ecology projects would also enable tests of commonly used surrogates of dispersal. For example, an important question in the dispersal literature is: to what extent, in what circumstances and for which species does structural connectivity provide functional connectivity [192]? Answers to such questions are essential for testing assumptions that underpin many land planning methods [73], [75], [193]. To learn about dispersal, the problem will need to be approached by comparing knowledge acquired using several different methods.

Conservation biologists are often challenged with the demand for information to support decision making in the short term [194]. To meet such challenges effectively, our third suggested area for priority research is to address the value of different kinds of dispersal information for distinguishing among management options (“value of information”)[195], [196]. Should a government agency aiming to minimise impacts of development on wildlife movement invest in genetics, GPS tracking, mark-recapture and behavioural studies, vegetation mapping or a subset of these? Further, should the level of information on dispersal be aimed at understanding population means or the complexity of individual variation? Process-based models and optimisation approaches are now possible that take into account the costs of gathering information and the costs of making poor decisions [195], [197]. For example, Hudgens et al. [60] found that a simple model using geographic distance could reasonably predict patch occupancy of an endangered butterfly. However, compared with a model based on detailed dispersal data, the simple model was not as good at predicting occupancy in restored habitat because it failed to identify an important dispersal barrier. The cost of developing more complex models was small, but they proved important for making effective management decisions in a complex landscape [60].

Development of the “value of information” framework will help to avoid what may be cultural constraints to gathering and applying dispersal knowledge [141], [142]. We have suggested there may be a culture that routinely accepts surrogates for dispersal within the land-planning topic that could undermine motivation to gather empirical data. Similarly, there may be entrenched cultural constraints that maintain the taxonomic biases in research. A value of information approach provides a framework for challenging cultural norms for using surrogates or to focus on plants, mammals and birds. It would encourage the question: is it most cost effective to undertake another study of bird dispersal, or can management that best conserves biodiversity be identified by improving knowledge of the less studied taxa, such as reptiles, amphibians and invertebrates?

### Management and Policy

Three key implications arising from this review that conservation managers and policy makers should consider relate to (i) funding, (ii) biases in dispersal research and (iii) methods for gathering dispersal data.

First, we found that the same broad research questions are still being asked about dispersal in a conservation context, consistent with the observation that the same threats to biodiversity are still present. The implication is that management effort (a product of policy direction and funding levels) has been inadequate to resolve the threats. Taking a particular focus on funding for dispersal research, how, when and where to fund dispersal research needs to be part of a balanced and strategic research portfolio. There are other knowledge gaps besides dispersal which need to be addressed because they too limit informed management choices.

Policy makers need also consider how to address the biases in research effort. Strong taxonomic biases identified in this and most other reviews mean that conservation policy is based on information about vascular plants, birds and some mammals. There were also biases among topics with climate change having orders of magnitude more research attention than invasive species. However, invasive species are among the top threats to biodiversity [198]. Switching funding priorities to counter key biases in the literature may help the knowledge base to represent biodiversity and threats more broadly, informing more inclusive conservation policies.

For managers, closing the dispersal knowledge gap will require judicious choices regarding how to gather dispersal information, considering the quality and limitations of alternative approaches. Expert opinion might best be avoided, surrogates such as corridors of vegetation need to be validated, easy to get occupancy data may have limited predictive value, and new genetic methods, while offering promise, are not a panacea. Therefore best practice is to take an experimental approach that compares alternative methods while also weighing up the kind of dispersal information that is needed to distinguish among management options.

## Conclusions

We confirmed that dispersal remains one of the pre-eminent knowledge gaps in conservation. Dispersal knowledge was widely recognised as limited 10–20 years ago, and it remains a significant limitation today. The dispersal knowledge gap has very serious consequences. Limited dispersal knowledge often makes it impossible to delineate the relative benefits of alternative management options. Consequently, despite substantial investment in land planning, restoration or other fields, the conservation benefit of implementing management will often remain unpredictable. This puts managers in the unenviable position of having increasingly sophisticated methods for making conservation decisions [199]–[201], but having an inadequate data base with which to make the tools work.

Although limitations of dispersal knowledge are commonly recognised, and although it can have important consequences for biodiversity management, there has been inadequate work to fill that knowledge gap. Presently, similar research questions are being asked, generally using the same methods as research from the 1990s. Further, those methods are usually indirect and are usually of low quality. Despite development of new ecological and genetic methods for measuring dispersal, these have not yet been widely adopted.

To help fill knowledge gaps about dispersal we suggest that more sophisticated programs of research into dispersal are needed that: (a) embrace new technologies and analytical approaches including adoption of a movement ecology approach; (b) help us to understand the complementarity and potential surrogacy of different methods for measuring dispersal; and, (c) are undertaken in a “value of information” framework so that we can discover how to gather the right dispersal knowledge to support particular management decisions. Ambitious, multi-species, multi-methodological programs of research are needed to solve these problems, and such future programs have the potential to generate general guidelines for the collection and application of dispersal information.

## Supporting Information

Appendix S1 **Papers used in the systematic review.**
(DOCX)

Appendix S2 **Risk of bias from special issues, commonly studied species or prolific authors**
(DOCX)

Table S1 **A description of the eighteen response variables that formed the basis of our review.**
(DOCX)

Table S2 **Test statistics from all analyses.**
(DOCX)

Table S3 **Summary of consistent trends across topics and times.**
(DOCX)

Table S4 **Number of papers in which dispersal data was gathered using particular methods.**
(DOCX)

Checklist S1 **PRISMA Checklist.**
(DOC)
