# Supplementary material for: The Trajectory of Dispersal Research in Conservation Biology. Systematic Review
Source: PLoS One. 2014 Apr 17;9(4):e95053. doi: 10.1371/journal.pone.0095053 (PMC3990620; doi:10.1371/journal.pone.0095053)
Supplement: Appendix S1 — Papers used in the systematic review. (DOCX) [file pone.0095053.s001.docx]

**Appendix S1.** The 478 papers used in the review.

Abbott I (1992) Biogeography of grasses (poaceae) on islands of southwestern australia. Australian Journal of Ecology 17: 289-296.

Abbott LK, Robson AD (1991) Factors influencing the occurrence of vesicular arbuscular mycorrhizas. Agriculture Ecosystems & Environment 35: 121-150.

Ackerly DD, Loarie SR, Cornwell WK, Weiss SB, Hamilton H, et al. (2010) The geography of climate change: Implications for conservation biogeography. Diversity and Distributions 16: 476-487.

Adler FR, Nuernberger B (1994) Persistence in patchy irregular landscapes. Theoretical Population Biology 45: 41-75.

Aiello-Lammens ME, Chu-Agor ML, Convertino M, Fischer RA, Linkov I, et al. (2011) The impact of sea-level rise on snowy plovers in florida: Integrating geomorphological, habitat, and metapopulation models. Global Change Biology 17: 3644-3654.

Akcakaya HR, Atwood JL (1997) A habitat based metapopulation model of the california gnatcatcher. Conservation Biology 11: 422-434.

Akcakaya HR, Baur B (1996) Effects of population subdivision and catastrophes on the persistence of a land snail metapopulation. Oecologia 105: 475-483.

Allan Green TG, Brabyn L, Beard C, Sancho LG (2012) Extremely low lichen growth rates in taylor valley, dry valleys, continental antarctica. Polar Biology 35: 535-541.

Almberg ES, Cross PC, Smith DW (2010) Persistence of canine distemper virus in the greater yellowstone ecosystem's carnivore community. Ecological Applications 20: 2058-2074.

Aminzadeh B, Khansefid M (2010) Improving the natural and built ecological systems in an urban environment. International Journal of Environmental Research 4: 361-372.

Andersen MC, Mahato D (1995) Demographic-models and reserve designs for the california spotted owl. Ecological Applications 5: 639-647.

Andow DA (1994) Community response to transgenic plant release - using mathematical-theory to predict effects of transgenic plants. Molecular Ecology 3: 65-70.

Armstrong DP, Wittmer HU (2011) Incorporating allee effects into reintroduction strategies. Ecological Research 26: 687-695.

Ashworth AC (1996) The response of arctic carabidae (coleoptera) to climate change based on the fossil record of the quaternary period. Annales Zoologici Fennici 33: 125-131.

Atzeni MG, Mayer DG, Spradbery JP, Anaman KA, Butler DG (1994) Comparison of the predicted impact of a screwworm fly outbreak in australia using a growth index model and a life-cycle model. Medical and Veterinary Entomology 8: 281-291.

Baasch A, Kirmer A, Tischew S (2012) Nine years of vegetation development in a postmining site: Effects of spontaneous and assisted site recovery. Journal of Applied Ecology 49: 251-260.

Baig MN (1992) Natural revegetation of coal-mine spoils in the rocky-mountains of alberta and its significance for species selection in land restoration. Mountain Research and Development 12: 285-300.

Bakker JP, Poschlod P, Strykstra RJ, Bekker RM, Thompson K (1996) Seed banks and seed dispersal: Important topics in restoration ecology. Acta Botanica Neerlandica 45: 461-490.

Barbet-Massin M, Thuiller W, Jiguet F (2012) The fate of european breeding birds under climate, land-use and dispersal scenarios. Global Change Biology 18: 881-890.

Barbour E, Kueppers LM (2012) Conservation and management of ecological systems in a changing california. Climatic Change 111: 135-163.

Barrett GW, Peles JD (1994) Optimizing habitat fragmentation - an agrolandscape perspective. Landscape and Urban Planning 28: 99-105.

Barrows CW, Fleming KD, Allen MF (2011) Identifying habitat linkages to maintain connectivity for corridor dwellers in a fragmented landscape. Journal of Wildlife Management 75: 682-691.

Barton HD, Wisely SM (2012) Phylogeography of striped skunks (mephitis mephitis) in north america: Pleistocene dispersal and contemporary population structure. Journal of Mammalogy 93: 38-51.

Barton KA, Hovestadt T, Phillips BL, Travis JMJ (2012) Risky movement increases the rate of range expansion. Proceedings of the Royal Society B-Biological Sciences 279: 1194-1202.

Baschak LA, Brown RD (1995) An ecological framework for the planning, design and management of urban river greenways. Landscape and Urban Planning 33: 211-225.

Bastin L, Thomas CD (1999) The distribution of plant species in urban vegetation fragments. Landscape Ecology 14: 493-507.

Bauer DM, Swallow SK, Paton PWC (2010) Cost-effective species conservation in exurban communities: A spatial analysis. Resource and Energy Economics 32: 180-202.

Beavon MA, Kelly D (2012) Invasional meltdown: Pollination of the invasive liana passiflora tripartita var. Mollissima (passifloraceae) in new zealand. New Zealand Journal of Ecology 36: 100-107.

Beger M, Linke S, Watts M, Game E, Treml E, et al. (2010) Incorporating asymmetric connectivity into spatial decision making for conservation. Conservation Letters 3: 359-368.

Beier P, Brost B (2010) Use of land facets to plan for climate change: Conserving the arenas, not the actors. Conservation Biology 24: 701-710.

Beissinger SR, Westphal MI (1998) On the use of demographic models of population viability in endangered species management. Journal of Wildlife Management 62: 821-841.

Beltman B, vandenBroek T, vanMaanen K, Vaneveld K (1996) Measures to develop a rich-fen wetland landscape with a full range of successional stages. Ecological Engineering 7: 299-313.

Bennett AF, Henein K, Merriam G (1994) Corridor use and the elements of corridor quality - chipmunks and fencerows in a farmland mosaic. Biological Conservation 68: 155-165.

Benningertruax M, Vankat JL, Schaefer RL (1992) Trail corridors as habitat and conduits for movement of plant-species in rocky-mountain-national-park, colorado, USA. Landscape Ecology 6: 269-278.

Bertiller MB, Coronato F (1994) Seed bank patterns of festuca-pallescens in semiarid patagonia (argentina) - a possible limit to bunch reestablishment. Biodiversity and Conservation 3: 57-67.

Bianconi GV, Suckow UMS, Cruz-Neto AP, Mikich SB (2012) Use of fruit essential oils to assist forest regeneration by bats. Restoration Ecology 20: 211-217.

Bode M, Brennan KEC (2011) Using population viability analysis to guide research and conservation actions for australia's threatened malleefowl leipoa ocellata. Oryx 45: 513-521.

Bokdam J, Devries MFW (1992) Forage quality as a limiting factor for cattle grazing in isolated dutch nature-reserves. Conservation Biology 6: 399-408.

Bossard CC (1991) The role of habitat disturbance, seed predation and ant dispersal on establishment of the exotic shrub cytisus-scoparius in california. American Midland Naturalist 126: 1-13.

Boutin C, Dobbie T, Carpenter D, Hebert CE (2011) Effects of double-crested cormorants (phalacrocorax auritus less.) on island vegetation, seedbank, and soil chemistry: Evaluating island restoration potential. Restoration Ecology 19: 720-727.

Bowers MA, Harris LC (1994) A large-scale metapopulation model of interspecific competition and environmental-change. Ecological Modelling 72: 251-273.

Boyero L, Pearson RG, Dudgeon D, Ferreira V, Graca MAS, et al. (2012) Global patterns of stream detritivore distribution: Implications for biodiversity loss in changing climates. Global Ecology and Biogeography 21: 134-141.

Boys CA, Kroon FJ, Glasby TM, Wilkinson K (2012) Improved fish and crustacean passage in tidal creeks following floodgate remediation. Journal of Applied Ecology 49: 223-233.

Bradstock RA, Bedward M, Scott J, Keith DA (1996) Simulation of the effect of spatial and temporal variation in fire regimes on the population viability of a banksia species. Conservation Biology 10: 776-784.

Brandt CA, Rickard WH (1994) Alien taxa in the north-american shrub-steppe 4 decades after cessation of livestock grazing and cultivation agriculture. Biological Conservation 68: 95-105.

Bridle JR, Polechova J, Kawata M, Butlin RK (2010) Why is adaptation prevented at ecological margins? New insights from individual-based simulations. Ecology Letters 13: 485-494.

Brodie J, Post E, Laurance WF (2012) Climate change and tropical biodiversity: A new focus. Trends in Ecology & Evolution 27: 145-150.

Brodie JF, Aslan CE (2012) Halting regime shifts in floristically intact tropical forests deprived of their frugivores. Restoration Ecology 20: 153-157.

Brook BW, Kikkawa J (1998) Examining threats faced by island birds: A population viability analysis on the capricorn silvereye using longterm data. Journal of Applied Ecology 35: 491-503.

Brothers TS, Spingarn A (1992) Forest fragmentation and alien plant invasion of central indiana old-growth forests. Conservation Biology 6: 91-100.

Buckley J, Butlin RK, Bridle JR (2012) Evidence for evolutionary change associated with the recent range expansion of the british butterfly, aricia agestis, in response to climate change. Molecular Ecology 21: 267-280.

Buma B, Wessman CA (2012) Differential species responses to compounded perturbations and implications for landscape heterogeneity and resilience. Forest Ecology and Management 266: 25-33.

Burley JB (1995) International greenways - a red-river valley case-study. Landscape and Urban Planning 33: 195-210.

Burns C, Sauer J (1992) Resistance by natural vegetation in the san gabriel mountains of california to invasion by introduced conifers. Global Ecology and Biogeography Letters 2: 46-51.

Butt KR, Frederickson J, Morris RM (1995) An earthworm cultivation and soil inoculation technique for land restoration. Ecological Engineering 4: 1-9.

Cabral JS, Bond WJ, Midgley GF, Rebelo AG, Thuiller W, et al. (2011) Effects of harvesting flowers from shrubs on the persistence and abundance of wild shrub populations at multiple spatial extents. Conservation Biology 25: 73-84.

Cairney JWG (2012) Extramatrical mycelia of ectomycorrhizal fungi as moderators of carbon dynamics in forest soil. Soil Biology & Biochemistry 47: 198-208.

Campbell C, Yang S, Albert R, Shea K (2011) A network model for plant-pollinator community assembly. Proceedings of the National Academy of Sciences of the United States of America 108: 197-202.

Cardoso P, Borges PAV, Triantis KA, Ferrandez MA, Martin JL (2011) Adapting the iucn red list criteria for invertebrates. Biological Conservation 144: 2432-2440.

Carey PD (1996) Disperse: A cellular automaton for predicting the distribution of species in a changed climate. Global Ecology and Biogeography Letters 5: 217-226.

Carroll C, Dunk JR, Moilanen A (2010) Optimizing resiliency of reserve networks to climate change: Multispecies conservation planning in the pacific northwest, USA. Global Change Biology 16: 891-904.

Carson HS, Cook GS, Lopez-Duarte PC, Levin LA (2011) Evaluating the importance of demographic connectivity in a marine metapopulation. Ecology 92: 1972-1984.

Catling PM, Porebski ZS (1994) The history of invasion and current status of glossy buckthorn, rhamnus-frangula, in southern ontario. Canadian Field-Naturalist 108: 305-310.

Cerdeira JO, Pinto LS, Cabeza M, Gaston KJ (2010) Species specific connectivity in reserve-network design using graphs. Biological Conservation 143: 408-415.

ChadwickFurman NE (1996) Reef coral diversity and global change. Global Change Biology 2: 559-568.

Chambers JC, Macmahon JA (1994) A day in the life of a seed - movements and fates of seeds and their implications for natural and managed systems. Annual Review of Ecology and Systematics 25: 263-292.

Chapman MG (2012) Restoring intertidal boulder-fields as habitat for "specialist" and "generalist" animals. Restoration Ecology 20: 277-285.

Chapple DG, Simmonds SM, Wong BBM (2012) Can behavioral and personality traits influence the success of unintentional species introductions? Trends in Ecology & Evolution 27: 57-64.

Chavez-Ramirez F, Wehtje W (2012) Potential impact of climate change scenarios on whooping crane life history. Wetlands 32: 11-20.

Chen H, Wang RQ, Ge XL, Zhang J, Du N, et al. (2012) Competition and soil fungi affect the physiological and growth traits of an alien and a native tree species. Photosynthetica 50: 77-85.

Chettri N (2010) Cross-taxon congruence in a trekking corridor of sikkim himalayas: Surrogate analysis for conservation planning. Journal for Nature Conservation 18: 75-88.

Chu-Agor ML, Munoz-Carpena R, Kiker GA, Aiello-Lammens ME, Akcakaya HR, et al. (2012) Simulating the fate of florida snowy plovers with sea-level rise: Exploring research and management priorities with a global uncertainty and sensitivity analysis perspective. Ecological Modelling 224: 33-47.

Cobben MMP, Verboom J, Opdam PFM, Hoekstra RF, Jochem R, et al. (2012) Landscape prerequisites for the survival of a modelled metapopulation and its neutral genetic diversity are affected by climate change. Landscape Ecology 27: 227-237.

Cohen AS (1995) Paleoecological approaches to the conservation biology of benthos in ancient lakes: A case study from lake tanganyika. Journal of the North American Benthological Society 14: 654-668.

Cohen JB, Gratto-Trevor C (2011) Survival, site fidelity, and the population dynamics of piping plovers in saskatchewan. Journal of Field Ornithology 82: 379-394.

Colchero F, Conde DA, Manterola C, Chavez C, Rivera A, et al. (2011) Jaguars on the move: Modeling movement to mitigate fragmentation from road expansion in the mayan forest. Animal Conservation 14: 158-166.

Collinge SK (1998) Spatial arrangement of habitat patches and corridors: Clues from ecological field experiments. Landscape and Urban Planning 42: 157-168.

Comeau LA, Sonier R, Hanson JM (2012) Seasonal movements of atlantic rock crab (cancer irroratus say) transplanted into a mussel aquaculture site. Aquaculture Research 43: 509-517.

Connor EF (1991) Colonization, survival, and causes of mortality of cameraria-hamadryadella (lepidoptera, gracillariidae) on 4 species of host plants. Ecological Entomology 16: 315-322.

Corbin JD, Holl KD (2012) Applied nucleation as a forest restoration strategy. Forest Ecology and Management 265: 37-46.

Cordell JR, Morgan CA, Simenstad CA (1992) Occurrence of the asian calanoid copepod pseudodiaptomus-inopinus in the zooplankton of the columbia river estuary. Journal of Crustacean Biology 12: 260-269.

Corti P, Shafer ABA, Coltman DW, Festa-Bianchet M (2011) Past bottlenecks and current population fragmentation of endangered huemul deer (hippocamelus bisulcus): Implications for preservation of genetic diversity. Conservation Genetics 12: 119-128.

Crespo-Perez V, Rebaudo F, Silvain J-F, Dangles O (2011) Modeling invasive species spread in complex landscapes: The case of potato moth in ecuador. Landscape Ecology 26: 1447-1461.

Cuda JP, Christ LR, Manrique V, Overholt WA, Wheeler GS, et al. (2012) Role of molecular genetics in identifying 'fine tuned' natural enemies of the invasive brazilian peppertree, schinus terebinthifolius: A review. Biocontrol 57: 227-233.

Cushman SA, Shirk A, Landguth EL (2012) Separating the effects of habitat area, fragmentation and matrix resistance on genetic differentiation in complex landscapes. Landscape Ecology 27: 369-380.

Daily GC, Ehrlich PR (1995) Preservation of biodiversity in small rain-forest patches - rapid evaluations using butterfly trapping. Biodiversity and Conservation 4: 35-55.

Davis WP, Taylor DS, Turner BJ (1995) Does the autecology of the mangrove rivulus fish (rivulus-marmoratus) reflect a paradigm for mangrove ecosystem sensitivity. Bulletin of Marine Science 57: 208-214.

Davy AJ, Brown MJH, Mossman HL, Grant A (2011) Colonization of a newly developing salt marsh: Disentangling independent effects of elevation and redox potential on halophytes. Journal of Ecology 99: 1350-1357.

De Blasio FV (1998) Diversity variation in isolated environments: Species-area effects from a stochastic model. Ecological Modelling 111: 93-98.

De Jager NR, Rohweder JJ (2012) Spatial patterns of aquatic habitat richness in the upper mississippi river floodplain, USA. Ecological Indicators 13: 275-283.

de la Montana E, Rey Benayas JM, Vasques A, Razola I, Cayuela L (2011) Conservation planning of vertebrate diversity in a mediterranean agricultural-dominant landscape. Biological Conservation 144: 2468-2478.

de Mendoza G, Rico E, Catalan J (2012) Predation by introduced fish constrains the thermal distribution of aquatic coleoptera in mountain lakes. Freshwater Biology 57: 803-814.

Debussche M, Lepart J (1992) Establishment of woody-plants in mediterranean old fields - opportunity in space and time. Landscape Ecology 6: 133-145.

Decout S, Manel S, Miaud C, Luque S (2012) Integrative approach for landscape-based graph connectivity analysis: A case study with the common frog (rana temporaria) in human-dominated landscapes. Landscape Ecology 27: 267-279.

Delavenne J, Metcalfe K, Smith RJ, Vaz S, Martin CS, et al. (2012) Systematic conservation planning in the eastern english channel: Comparing the marxan and zonation decision-support tools. Ices Journal of Marine Science 69: 75-83.

Dennison S, Smith SM, Stow AJ (2012) Long-distance geneflow and habitat specificity of the rock-dwelling coppertail skink, ctenotus taeniolatus. Austral Ecology 37: 258-267.

Depietri DE (1992) Alien shrubs in a national-park - can they help in the recovery of natural degraded forest. Biological Conservation 62: 127-130.

Diaz FP, Latorre C, Maldonado A, Quade J, Betancourt JL (2012) Rodent middens reveal episodic, long-distance plant colonizations across the hyperarid atacama desert over the last 34,000 years. Journal of Biogeography 39: 510-525.

Diekmann OE, Serrao EA (2012) Range-edge genetic diversity: Locally poor extant southern patches maintain a regionally diverse hotspot in the seagrass zostera marina. Molecular Ecology 21: 1647-1657.

Diekoetter T, Baveco H, Arens P, Rothenbuehler C, Billeter R, et al. (2010) Patterns of habitat occupancy, genetic variation and predicted movement of a flightless bush cricket, pholidoptera griseoaptera, in an agricultural mosaic landscape. Landscape Ecology 25: 449-461.

Dobrovolski R, Melo AS, Cassemiro FAS, Felizola Diniz-Filho JA (2012) Climatic history and dispersal ability explain the relative importance of turnover and nestedness components of beta diversity. Global Ecology and Biogeography 21: 191-197.

Doney SC, Ruckelshaus M, Duffy JE, Barry JP, Chan F, et al. (2012) Climate change impacts on marine ecosystems. In: Carlson CAGSJ, editor. Annual review of marine science, vol 4. pp. 11-37.

Douda K, Vrtilek M, Slavik O, Reichard M (2012) The role of host specificity in explaining the invasion success of the freshwater mussel anodonta woodiana in europe. Biological Invasions 14: 127-137.

Dovrat G, Perevolotsky A, Ne'eman G (2012) Wild boars as seed dispersal agents of exotic plants from agricultural lands to conservation areas. Journal of Arid Environments 78: 49-54.

Drechsler M, Wissel C (1998) Trade-offs between local and regional scale management of metapopulations. Biological Conservation 83: 31-41.

Dudley TL, Bean DW (2012) Tamarisk biocontrol, endangered species risk and resolution of conflict through riparian restoration. Biocontrol 57: 331-347.

Duputie A, Massol F, Chuine I, Kirkpatrick M, Ronce O (2012) How do genetic correlations affect species range shifts in a changing environment? Ecology Letters 15: 251-259.

Dyer C, Richardson DM (1992) Population-genetics of the invasive australian shrub hakea-sericea (proteaceae) in south-africa. South African Journal of Botany 58: 117-124.

Dyke AS, Hooper J, Savelle JM (1996) A history of sea ice in the canadian arctic archipelago based on postglacial remains of the bowhead whale (balaena mysticetus). Arctic 49: 235-255.

Edwards HJ, Elliott IA, Pressey RL, Mumby PJ (2010) Incorporating ontogenetic dispersal, ecological processes and conservation zoning into reserve design. Biological Conservation 143: 457-470.

Ehmann WJ, MacMahon JA (1996) Initial tests for priority effects among spiders that co-occur on sagebrush shrubs. Journal of Arachnology 24: 173-185.

Ellsworth DL, Honeycutt RL, Silvy NJ, Smith MH, Bickham JW, et al. (1994) White-tailed deer restoration to the southeastern united-states - evaluating genetic-variation. Journal of Wildlife Management 58: 686-697.

Elosegi A, Flores L, Diez J (2011) The importance of local processes on river habitat characteristics: A basque stream case study. Limnetica 30: 183-196.

Equihua M, Usher MB (1993) Impact of carpets of the invasive moss campylopus-introflexus on calluna-vulgaris regeneration. Journal of Ecology 81: 359-365.

Eros T, Schmera D, Schick RS (2011) Network thinking in riverscape conservation - a graph-based approach. Biological Conservation 144: 184-192.

Esselman PC, Allan JD (2011) Application of species distribution models and conservation planning software to the design of a reserve network for the riverine fishes of northeastern mesoamerica. Freshwater Biology 56: 71-88.

Feeley KJ (2012) Distributional migrations, expansions, and contractions of tropical plant species as revealed in dated herbarium records. Global Change Biology 18: 1335-1341.

Fernandez-Chacon A, Bertolero A, Amengual A, Tavecchia G, Homar V, et al. (2011) Spatial heterogeneity in the effects of climate change on the population dynamics of a mediterranean tortoise. Global Change Biology 17: 3075-3088.

Fimbel RA, Fimbel CC (1996) The role of exotic conifer plantations in rehabilitating degraded tropical forest lands: A case study from the kibale forest in uganda. Forest Ecology and Management 81: 215-226.

Fischer SF, Poschlod P, Beinlich B (1996) Experimental studies on the dispersal of plants and animals on sheep in calcareous grasslands. Journal of Applied Ecology 33: 1206-1222.

Flores L, Larranaga A, Diez J, Elosegi A (2011) Experimental wood addition in streams: Effects on organic matter storage and breakdown. Freshwater Biology 56: 2156-2167.

Fordham DA, Akcakaya HR, Araujo MB, Elith J, Keith DA, et al. (2012) Plant extinction risk under climate change: Are forecast range shifts alone a good indicator of species vulnerability to global warming? Global Change Biology 18: 1357-1371.

Forys EA, Humphrey SR (1999) Use of population viability analysis to evaluate management options for the endangered lower keys marsh rabbit. Journal of Wildlife Management 63: 251-260.

Foster DR, Zebryk TM (1993) Long-term vegetation dynamics and disturbance history of a tsuga-dominated forest in new-england. Ecology 74: 982-998.

Frank K, Wissel C (1998) Spatial aspects of metapopulation survival - from model results to rules of thumb for landscape management. Landscape Ecology 13: 363-379.

Frei ES, Scheepens JF, Stoecklin J (2012) Dispersal and microsite limitation of a rare alpine plant. Plant Ecology 213: 395-406.

Fries C, Carlsson M, Dahlin B, Lamas T, Sallnas O (1998) A review of conceptual landscape planning models for multiobjective forestry in sweden. Canadian Journal of Forest Research-Revue Canadienne De Recherche Forestiere 28: 159-167.

Fry G, SarlovHerlin I (1997) The ecological and amenity functions of woodland edges in the agricultural landscape, a basis for design and management. Landscape and Urban Planning 37: 45-55.

Fuller JL (1997) Holocene forest dynamics in southern ontario, canada: Fine-resolution pollen data. Canadian Journal of Botany-Revue Canadienne De Botanique 75: 1714-1727.

Galatowitsch SM, vanderValk AG (1996) Vegetation and environmental conditions in recently restored wetlands in the prairie pothole region of the USA. Vegetatio 126: 89-99.

Galatowitsch SM, vanderValk AG (1996) The vegetation of restored and natural prairie wetlands. Ecological Applications 6: 102-112.

Galen C, Stanton ML (1995) Responses of snowbed plant-species to changes in growing-season length. Ecology 76: 1546-1557.

Game ET, Lipsett-Moore G, Saxon E, Peterson N, Sheppard S (2011) Incorporating climate change adaptation into national conservation assessments. Global Change Biology 17: 3150-3160.

Gangloff MM, Hartfield EE, Werneke DC, Feminella JW (2011) Associations between small dams and mollusk assemblages in alabama streams. Journal of the North American Benthological Society 30: 1107-1116.

Gaona P, Ferreras P, Delibes M (1998) Dynamics and viability of a metapopulation of the endangered iberian lynx (lynx pardinus). Ecological Monographs 68: 349-370.

Garcia-Feced C, Saura S, Elena-Rossello R (2011) Improving landscape connectivity in forest districts: A two-stage process for prioritizing agricultural patches for reforestation. Forest Ecology and Management 261: 154-161.

Gargan PG, Roche WK, Keane S, King JJ, Cullagh A, et al. (2011) Comparison of field- and gis-based assessments of barriers to atlantic salmon migration: A case study in the nore catchment, republic of ireland. Journal of Applied Ichthyology 27: 66-72.

Geml J, Kauff F, Brochmann C, Lutzoni F, Laursen GA, et al. (2012) Frequent circumarctic and rare transequatorial dispersals in the lichenised agaric genus lichenomphalia (hygrophoraceae, basidiomycota). Fungal Biology 116: 388-400.

Geremia C, White PJ, Wallen RL, Watson FGR, Treanor JJ, et al. (2011) Predicting bison migration out of yellowstone national park using bayesian models. PloS one 6.

Gibson CWD, Brown VK (1991) The nature and rate of development of calcareous grassland in southern britain. Biological Conservation 58: 297-316.

Gilman E, Dunn D, Read A, Hyrenbach KD, Warner R (2011) Designing criteria suites to identify discrete and networked sites of high value across manifestations of biodiversity. Biodiversity and Conservation 20: 3363-3383.

Gimmi U, Lachat T, Buergi M (2011) Reconstructing the collapse of wetland networks in the swiss lowlands 1850-2000. Landscape Ecology 26: 1071-1083.

Glen AS, Byrom AE, Pech RP, Cruz J, Schwab A, et al. (2012) Ecology of brushtail possums in a new zealand dryland ecosystem. New Zealand Journal of Ecology 36: 29-37.

Goodwin JR, Doescher PS, Eddleman LE (1995) After-ripening in festuca-idahoensis seeds - adaptive dormancy and implications for restoration. Restoration Ecology 3: 137-142.

Gravel D, Canard E, Guichard F, Mouquet N (2011) Persistence increases with diversity and connectance in trophic metacommunities. PloS one 6: e19374.

Gray DK, Arnott SE (2011) The interplay between environmental conditions and allee effects during the recovery of stressed zooplankton communities. Ecological Applications 21: 2652-2663.

Greenslade P, Convey P (2012) Exotic collembola on subantarctic islands: Pathways, origins and biology. Biological Invasions 14: 405-417.

Grist EPM (1999) The significance of spatio-temporal neighbourhood on plant competition for light and space. Ecological Modelling 121: 63-78.

Gurrutxaga M, Rubio L, Saura S (2011) Key connectors in protected forest area networks and the impact of highways: A transnational case study from the cantabrian range to the western alps (sw europe). Landscape and Urban Planning 101: 310-320.

Hall KT, Baker MB, Hadfield MG (2010) Using dispersal rates to guide translocation across impermeable wildlife reserve boundaries: Hawaiian tree snails as a practical example. Malacologia 52: 67-80.

Halpin PN (1997) Global climate change and natural-area protection: Management responses and research directions. Ecological Applications 7: 828-843.

Hammill E, Curtis JMR, Patterson DA, Farrell AP, Sierocinski T, et al. (2012) Comparison of techniques for correlating survival and gene expression data from wild salmon. Ecology of Freshwater Fish 21: 189-199.

Hanak E, Moreno G (2012) California coastal management with a changing climate. Climatic Change 111: 45-73.

Hanski I (1994) A practical model of metapopulation dynamics. Journal of Animal Ecology 63: 151-162.

Hart AT, Hilton MJ, Wakes SJ, Dickinson KJM (2012) The impact of ammophila arenaria foredune development on downwind aerodynamics and parabolic dune development. Journal of Coastal Research 28: 112-122.

Hartvigsen G, Levin S (1997) Evolution and spatial structure interact to influence plant-herbivore population and community dynamics. Proceedings of the Royal Society of London Series B-Biological Sciences 264: 1677-1685.

Hayasaka D, Akasaka M, Miyauchi D, Box EO, Uchida T (2012) Qualitative variation in roadside weed vegetation along an urban-rural road gradient. Flora 207: 126-132.

Heelemann S, Krug CB, Esler KJ, Reisch C, Poschlod P (2012) Pioneers and perches-promising restoration methods for degraded renosterveld habitats? Restoration Ecology 20: 18-23.

Heijnis CE, Lombard AT, Cowling RM, Desmet PG (1999) Picking up the pieces: A biosphere reserve framework for a fragmented landscape - the coastal lowlands of the western cape, south africa. Biodiversity and Conservation 8: 471-496.

Hemptinne JL, Magro A, Evans EW, Dixon AFG (2012) Body size and the rate of spread of invasive ladybird beetles in north america. Biological Invasions 14: 595-605.

Hendrix WH, Showers WB (1992) Tracing black cutworm and armyworm (lepidoptera, noctuidae) northward migration using pithecellobium-and calliandra pollen. Environmental Entomology 21: 1092-1096.

Henein K, Wegner J, Merriam G (1998) Population effects of landscape model manipulation on two behaviourally different woodland small mammals. Oikos 81: 168-186.

Hensen I, Cierjacks A, Hirsch H, Kessler M, Romoleroux K, et al. (2012) Historic and recent fragmentation coupled with altitude affect the genetic population structure of one of the world's highest tropical tree line species. Global Ecology and Biogeography 21: 455-464.

Herben T (1994) Local-rate of spreading and patch dynamics of an invasive moss species, orthodontium lineare. Journal of Bryology 18: 115-125.

Herlin ILS, Fry GLA (2000) Dispersal of woody plants in forest edges and hedgerows in a southern swedish agricultural area: The role of site and landscape structure. Landscape Ecology 15: 229-242.

Hermoso V, Linke S, Prenda J, Possingham HP (2011) Addressing longitudinal connectivity in the systematic conservation planning of fresh waters. Freshwater Biology 56: 57-70.

Herwitz SR (1992) Quaternary vegetation change and dune formation on bermuda - a discussion. Global Ecology and Biogeography Letters 2: 65-70.

Hester S, Cacho O (2012) Optimization of search strategies in managing biological invasions: A simulation approach. Human and Ecological Risk Assessment 18: 181-199.

Hietz P, Winkler M, Scheffknecht S, Huelber K (2012) Germination of epiphytic bromeliads in forests and coffee plantations: Microclimate and substrate effects. Biotropica 44: 197-204.

Hinch SG, Healey MC, Diewert RE, Thomson KA, Hourston R, et al. (1995) Potential effects of climate change on marine growth and survival of fraser river sockeye salmon. Canadian Journal of Fisheries and Aquatic Sciences 52: 2651-2659.

Hoagstrom CW, Brooks JE, Davenport SR (2011) A large-scale conservation perspective considering endemic fishes of the north american plains. Biological Conservation 144: 21-34.

Hobbs RJ, Norton DA (1996) Towards a conceptual framework for restoration ecology. Restoration Ecology 4: 93-110.

Hoelzel N, Buisson E, Dutoit T (2012) Species introduction - a major topic in vegetation restoration. Applied Vegetation Science 15: 161-165.

Hogg ID, Williams DD, Eadie JM, Butt SA (1995) The consequences of global warming for stream invertebrates - a field simulation. Journal of Thermal Biology 20: 199-206.

Holdo RM, Fryxell JM, Sinclair ARE, Dobson A, Holt RD (2011) Predicted impact of barriers to migration on the serengeti wildebeest population. PloS one 6.

Holland JD (2010) Isolating spatial effects on beta diversity to inform forest landscape planning. Landscape Ecology 25: 1349-1362.

Holliday JA, Suren H, Aitken SN (2012) Divergent selection and heterogeneous migration rates across the range of sitka spruce (picea sitchensis). Proceedings of the Royal Society B-Biological Sciences 279: 1675-1683.

Holmes PM (1990) Dispersal and predation in alien acacia. Oecologia 83: 288-290.

Holmes PM (1990) Dispersal and predation of alien acacia seeds - effects of season and invading stand density. South African Journal of Botany 56: 428-434.

Holusa J, Kocarek P, Marhoul P, Skokanova H (2012) Platycleis vittata (orthoptera: Tettigoniidae) in the northwestern part of its range is close to extinction: Is this the result of landscape changes? Journal of Insect Conservation 16: 295-303.

Horwood JW, Nichols JH, Milligan S (1998) Evaluation of closed areas for fish stock conservation. Journal of Applied Ecology 35: 893-903.

Hough-Goldstein J, Lake E, Reardon R (2012) Status of an ongoing biological control program for the invasive vine, persicaria perfoliata in eastern north america. Biocontrol 57: 181-189.

Hovick SM, Campbell LG, Snow AA, Whitney KD (2012) Hybridization alters early life-history traits and increases plant colonization success in a novel region. American Naturalist 179: 192-203.

Howe WH, Knopf FL (1991) On the imminent decline of rio-grande cottonwoods in central new-mexico. Southwestern Naturalist 36: 218-224.

Hsu RCC, Tamis WLM, Raes N, de Snoo GR, Wolf JHD, et al. (2012) Simulating climate change impacts on forests and associated vascular epiphytes in a subtropical island of east asia. Diversity and Distributions 18: 334-347.

Huntley B (1991) How plants respond to climate change - migration rates, individualism and the consequences for plant-communities. Annals of Botany 67: 15-22.

Huntley B (1995) Plant-species response to climate-change - implications for the conservation of european birds. Ibis 137: S127-S138.

Hurst TP, Moss JH, Miller JA (2012) Distributional patterns of 0-group pacific cod (gadus macrocephalus) in the eastern bering sea under variable recruitment and thermal conditions. Ices Journal of Marine Science 69: 163-174.

Ibrahim KM, Nichols RA, Hewitt GM (1996) Spatial patterns of genetic variation generated by different forms of dispersal during range expansion. Heredity 77: 282-291.

Ieronymidou C, Collar NJ, Dolman PM (2012) Endemic cyprus warbler sylvia melanothorax and colonizing sardinian warbler sylvia melanocephala show different habitat associations. Ibis 154: 248-259.

Jacobi MN, Jonsson PR (2011) Optimal networks of nature reserves can be found through eigenvalue perturbation theory of the connectivity matrix. Ecological Applications 21: 1861-1870.

Jacobs JD, Heron R, Luther JE (1993) Recent changes at the northwest margin of the barnes ice cap, baffin-island, nwt, canada. Arctic and Alpine Research 25: 341-352.

Jastrow JD, Miller RM (1993) Neighbor influences on root morphology and mycorrhizal fungus colonization in tallgrass prairie plants. Ecology 74: 561-569.

Jeltsch F, Moloney KA, Schwager M, Koerner K, Blaum N (2011) Consequences of correlations between habitat modifications and negative impact of climate change for regional species survival. Agriculture Ecosystems & Environment 145: 49-58.

Johnson WC, Adkisson CS, Crow TR, Dixon MD (1997) Nut caching by blue jays (cyanocitta cristata l.): Implications for tree demography. American Midland Naturalist 138: 357-370.

Johst K, Drechsler M, van Teeffelen AJA, Hartig F, Vos CC, et al. (2011) Biodiversity conservation in dynamic landscapes: Trade-offs between number, connectivity and turnover of habitat patches. Journal of Applied Ecology 48: 1227-1235.

Jollivet D, Chevaldonne P, Planque B (1999) Hydrothermal-vent alvinellid polychaete dispersal in the eastern pacific. 2. A metapopulation model based on habitat shifts. Evolution 53: 1128-1142.

Jourdan-Pineau H, David P, Crochet P-A (2012) Phenotypic plasticity allows the mediterranean parsley frog pelodytes punctatus to exploit two temporal niches under continuous gene flow. Molecular Ecology 21: 876-886.

Jud ZR, Layman CA (2012) Site fidelity and movement patterns of invasive lionfish, pterois spp., in a florida estuary. Journal of Experimental Marine Biology and Ecology 414: 69-74.

Judd KW, Mason CF (1995) Earthworm populations of a restored landfill site. Pedobiologia 39: 107-115.

Jump AS, Huang T-J, Chou C-H (2012) Rapid altitudinal migration of mountain plants in taiwan and its implications for high altitude biodiversity. Ecography 35: 204-210.

Kang Y, Armbruster D (2011) Dispersal effects on a discrete two-patch model for plant-insect interactions. Journal of Theoretical Biology 268: 84-97.

Karpa DM, Vitousek PM (1994) Successional development of a hawaiian montane grassland. Biotropica 26: 2-11.

Karsiotis SI, Pierce LR, Brown JE, Stepien CA (2012) Salinity tolerance of the invasive round goby: Experimental implications for seawater ballast exchange and spread to north american estuaries. Journal of Great Lakes Research 38: 121-128.

Kaufmann S, McKey DB, Hossaertmckey M, Horvitz CC (1991) Adaptations for a 2-phase seed dispersal system involving vertebrates and ants in a hemiepiphytic fig (ficus-microcarpa, moraceae). American Journal of Botany 78: 971-977.

Kelehear C, Brown GP, Shine R (2012) Rapid evolution of parasite life history traits on an expanding range-edge. Ecology Letters 15: 329-337.

Kemper J, Cowling RM, Richardson DM, Forsyth GG, McKelly DH (2000) Landscape fragmentation in south coast renosterveld, south africa, in relation to rainfall and topography. Austral Ecology 25: 179-186.

Kennedy AD (1995) Antarctic terrestrial ecosystem response to global environmental-change. Annual Review of Ecology and Systematics 26: 683-704.

Keppel G, Van Niel KP, Wardell-Johnson GW, Yates CJ, Byrne M, et al. (2012) Refugia: Identifying and understanding safe havens for biodiversity under climate change. Global Ecology and Biogeography 21: 393-404.

Kertson BN, Spencer RD, Marzluff JM, Hepinstall-Cymerman J, Grue CE (2011) Cougar space use and movements in the wildland-urban landscape of western washington. Ecological Applications 21: 2866-2881.

Kettle CJ, Ennos RA, Jaffre T, McCoy S, Le Borgne T, et al. (2012) Importance of demography and dispersal for the resilience and restoration of a critically endangered tropical conifer araucaria nemorosa. Diversity and Distributions 18: 248-259.

Khanna S, Santos MJ, Hestir EL, Ustin SL (2012) Plant community dynamics relative to the changing distribution of a highly invasive species, eichhornia crassipes: A remote sensing perspective. Biological Invasions 14: 717-733.

Kindvall O (1999) Dispersal in a metapopulation of the bush cricket, metrioptera bicolor (orthoptera : Tettigoniidae). Journal of Animal Ecology 68: 172-185.

King J, Moutsinga JB, Doufoulon G (1997) Conversion of anthropogenic savanna to production forest through fire-protection of the forest-savanna edge in gabon, central africa. Forest Ecology and Management 94: 233-247.

King T, Chamberlan C, Courage A (2012) Assessing initial reintroduction success in long-lived primates by quantifying survival, reproduction, and dispersal parameters: Western lowland gorillas (gorilla gorilla gorilla) in congo and gabon. International Journal of Primatology 33: 134-149.

Kininmonth S, Beger M, Bode M, Peterson E, Adams VM, et al. (2011) Dispersal connectivity and reserve selection for marine conservation. Ecological Modelling 222: 1272-1282.

Kininmonth S, Drechsler M, Johst K, Possingham HP (2010) Metapopulation mean life time within complex networks. Marine Ecology-Progress Series 417: 139-149.

Knaapen JP, Scheffer M, Harms B (1992) Estimating habitat isolation in landscape planning. Landscape and Urban Planning 23: 1-16.

Kornis MS, Mercado-Silva N, Vander Zanden MJ (2012) Twenty years of invasion: A review of round goby neogobius melanostomus biology, spread and ecological implications. Journal of Fish Biology 80: 235-285.

Kouki J, Hyvarinen E, Lappalainen H, Martikainen P, Simila M (2012) Landscape context affects the success of habitat restoration: Large-scale colonization patterns of saproxylic and fire-associated species in boreal forests. Diversity and Distributions 18: 348-355.

Kramer-Schadt S, Kaiser TS, Frank K, Wiegand T (2011) Analyzing the effect of stepping stones on target patch colonisation in structured landscapes for eurasian lynx. Landscape Ecology 26: 501-513.

Krankina ON, Dixon RK, Kirilenko AP, Kobak KI (1997) Global climate change adaptation: Examples from russian boreal forests. Climatic Change 36: 197-215.

Krauchi N, Kienast F (1993) Modeling sub-alpine forest dynamics as influenced by a changing environment. Water Air and Soil Pollution 68: 185-197.

Kremer A, Ronce O, Robledo-Arnuncio JJ, Guillaume F, Bohrer G, et al. (2012) Long-distance gene flow and adaptation of forest trees to rapid climate change. Ecology Letters 15: 378-392.

Krkosek M, Connors BM, Lewis MA, Poulin R (2012) Allee effects may slow the spread of parasites in a coastal marine ecosystem. American Naturalist 179: 401-412.

Kuaraksa C, Elliott S, Hossaert-Mckey M (2012) The phenology of dioecious ficus spp. Tree species and its importance for forest restoration projects. Forest Ecology and Management 265: 82-93.

Kubisch A, Poethke H-J (2011) Range border formation in a world with increasing climatic variance. Evolutionary Ecology Research 13: 159-169.

Lambertini C, Mendelssohn IA, Gustafsson MHG, Olesen B, Riis T, et al. (2012) Tracing the origin of gulf coast phragmites (poaceae): A story of long-distance dispersal and hybridization. American Journal of Botany 99: 538-551.

Lande R, Engen S, Saether BE (1999) Spatial scale of population synchrony: Environmental correlation versus dispersal and density regulation. American Naturalist 154: 271-281.

Landhausser SM, Wein RW (1993) Postfire vegetation recovery and tree establishment at the arctic treeline - climate-change vegetation-response hypotheses. Journal of Ecology 81: 665-672.

Landis DA, Fiedler AK, Hamm CA, Cuthrell DL, Schools EH, et al. (2012) Insect conservation in michigan prairie fen: Addressing the challenge of global change. Journal of Insect Conservation 16: 131-142.

Larsen TH (2012) Upslope range shifts of andean dung beetles in response to deforestation: Compounding and confounding effects of microclimatic change. Biotropica 44: 82-89.

Lauridsen TL, Jeppesen E, Sondergaard M (1994) Colonization and succession of submerged macrophytes in shallow lake vaeng during the 1st 5 years following fish manipulation. Hydrobiologia 275: 233-242.

Lavoie C, Payette S (1996) The long-term stability of the boreal forest limit in subarctic quebec. Ecology 77: 1226-1233.

Lawler JJ, Olden JD (2011) Reframing the debate over assisted colonization. Frontiers in Ecology and the Environment 9: 569-574.

Lawson DM, Regan HM, Zedler PH, Franklin A (2010) Cumulative effects of land use, altered fire regime and climate change on persistence of ceanothus verrucosus, a rare, fire-dependent plant species. Global Change Biology 16: 2518-2529.

Leathwick JR, Moilanen A, Ferrier S, Julian K (2010) Complementarity-based conservation prioritization using a community classification, and its application to riverine ecosystems. Biological Conservation 143: 984-991.

Legendre S, Clobert J, Moller AP, Sorci G (1999) Demographic stochasticity and social mating system in the process of extinction of small populations: The case of passerines introduced to new zealand. American Naturalist 153: 449-463.

Lei GC, Hanski I (1998) Spatial dynamics of two competing specialist parasitoids in a host metapopulation. Journal of Animal Ecology 67: 422-433.

Leimar O, Norberg U (1997) Metapopulation extinction and genetic variation in dispersal-related traits. Oikos 80: 448-458.

Leishman MR, Hughes L, French K, Armstrong D, Westoby M (1992) Seed and seedling biology in relation to modeling vegetation dynamics under global climate change. Australian Journal of Botany 40: 599-613.

Leishman MR, Westoby M (1992) Classifying plants into groups on the basis of associations of individual traits evidence from australian semiarid woodlands. Journal of Ecology 80: 417-424.

Leithead M, Anand M, Duarte LdS, Pillar VD (2012) Causal effects of latitude, disturbance and dispersal limitation on richness in a recovering temperate, subtropical and tropical forest. Journal of Vegetation Science 23: 339-351.

LeMaitre DC, VanWilgen BW, Chapman RA, McKelly DH (1996) Invasive plants and water resources in the western cape province, south africa: Modelling the consequences of a lack of management. Journal of Applied Ecology 33: 161-172.

Lengyel S, Varga K, Kosztyi B, Lontay L, Deri E, et al. (2012) Grassland restoration to conserve landscape-level biodiversity: A synthesis of early results from a large-scale project. Applied Vegetation Science 15: 264-276.

Lennon JJ, Turner JRG, Connell D (1997) A metapopulation model of species boundaries. Oikos 78: 486-502.

Letcher BH, Priddy JA, Walters JR, Crowder LB (1998) An individual-based, spatially-explicit simulation model of the population dynamics of the endangered red-cockaded woodpecker, picoides borealis. Biological Conservation 86: 1-14.

Levin LA, Talley D, Thayer G (1996) Succession of macrobenthos in a created salt marsh. Marine Ecology-Progress Series 141: 67-82.

Lindenmayer DB, McCarthy MA, Pope ML (1999) Arboreal marsupial incidence in eucalypt patches in south-eastern australia: A test of hanski's incidence function metapopulation model for patch occupancy. Oikos 84: 99-109.

Lindenmayer DB, Possingham HP (1996) Modelling the inter-relationships between habitat patchiness, dispersal capability and metapopulation persistence of the endangered species, leadbeater's possum, in south-eastern australia. Landscape Ecology 11: 79-105.

Linehan J, Gross M, Finn J (1995) Greenway planning - developing a landscape ecological network approach. Landscape and Urban Planning 33: 179-193.

Linke S, Turak E, Nel J (2011) Freshwater conservation planning: The case for systematic approaches. Freshwater Biology 56: 6-20.

Littlewood NA, Stewart AJA, Woodcock BA (2012) Science into practice - how can fundamental science contribute to better management of grasslands for invertebrates? Insect Conservation and Diversity 5: 1-8.

Litvaitis JA, Villafuerte R (1996) Factors affecting the persistence of new england cottontail metapopulations: The role of habitat management. Wildlife Society Bulletin 24: 686-693.

Llewellyn DW, Shaffer GP, Craig NJ, Creasman L, Pashley D, et al. (1996) A decision-support system for prioritizing restoration sites on the mississippi river alluvial plain. Conservation Biology 10: 1446-1455.

Lloret F, Escudero A, Maria Iriondo J, Martinez-Vilalta J, Valladares F (2012) Extreme climatic events and vegetation: The role of stabilizing processes. Global Change Biology 18: 797-805.

Locke CM, Rissman AR (2012) Unexpected co-benefits: Forest connectivity and property tax incentives. Landscape and Urban Planning 104: 418-425.

Loehle C, LeBlanc D (1996) Model-based assessments of climate change effects on forests: A critical review. Ecological Modelling 90: 1-31.

Lonsdale WM (1993) Rates of spread of an invading species - mimosa-pigra in northern australia. Journal of Ecology 81: 513-521.

Lopes PM, Caliman A, Carneiro LS, Bini LM, Esteves FA, et al. (2011) Concordance among assemblages of upland amazonian lakes and the structuring role of spatial and environmental factors. Ecological Indicators 11: 1171-1176.

Lorenz AW, Korte T, Sundermann A, Januschke K, Haase P (2012) Macrophytes respond to reach-scale river restorations. Journal of Applied Ecology 49: 202-212.

Lowday JE, Marrs RH (1992) Control of bracken and the restoration of heathland .3. Bracken litter disturbance and heathland restoration. Journal of Applied Ecology 29: 212-217.

Lu J, Wang H, Pan M, Xia J, Xing W, et al. (2012) Using sediment seed banks and historical vegetation change data to develop restoration criteria for a eutrophic lake in china. Ecological Engineering 39: 95-103.

MacDonald LA, Balasubramaniam AM, Hall RI, Wolfe BB, Sweetman JN (2012) Developing biomonitoring protocols for shallow arctic lakes using diatoms and artificial substrate samplers. Hydrobiologia 683: 231-248.

Mackie GL (1991) Biology of the exotic zebra mussel, dreissena-polymorpha, in relation to native bivalves and its potential impact in lake st-clair. Hydrobiologia 219: 251-268.

MacPherson JL, Bright PW (2011) Metapopulation dynamics and a landscape approach to conservation of lowland water voles (arvicola amphibius). Landscape Ecology 26: 1395-1404.

Majer JD (1996) Ant recolonization of rehabilitated bauxite mines at trombetas, para, brazil. Journal of Tropical Ecology 12: 257-273.

Malanson GP, Cramer BE (1999) Ants in labyrinths: Lessons for critical landscapes. Professional Geographer 51: 155-170.

Mangialajo L, Chiantore M, Susini M-L, Meinesz A, Cattaneo-Vietti R, et al. (2012) Zonation patterns and interspecific relationships of fucoids in microtidal environments. Journal of Experimental Marine Biology and Ecology 412: 72-80.

Marion SR, Orth RJ (2012) Seedling establishment in eelgrass: Seed burial effects on winter losses of developing seedlings. Marine Ecology-Progress Series 448: 197-207.

Markham A (1996) Potential impacts of climate change on ecosystems: A review of implications for policymakers and conservation biologists. Climate Research 6: 179-191.

Markle DF (2011) Size-structured spatial patterns as a measure of larval dispersal and emigration. Western North American Naturalist 71: 456-471.

Martinez-Duro E, Ferrandis P, Herranz JM, Copete MA (2010) Do seed harvesting ants threaten the viability of a critically endangered non-myrmecochorous perennial plant population? A complex interaction. Population Ecology 52: 397-405.

Mason CE, Romig RF, Wendel LE, Wood LA (1994) Distribution and abundance of larval parasitoids of european corn-borer (lepidoptera, pyralidae) in the east central united-states. Environmental Entomology 23: 521-531.

Mattsson BJ, Runge MC, Devries JH, Boomer GS, Eadie JM, et al. (2012) A modeling framework for integrated harvest and habitat management of north american waterfowl: Case-study of northern pintail metapopulation dynamics. Ecological Modelling 225: 146-158.

May SA, Norton TW (1996) Influence of fragmentation and disturbance on the potential impact of feral predators on native fauna in australian forest ecosystems. Wildlife Research 23: 387-400.

McCarthy MA, Lindenmayer DB (1999) Conservation of the greater glider (petauroides volans) in remnant native vegetation within exotic plantation forest. Animal Conservation 2: 203-209.

McCullough DG, Mercader RJ (2012) Evaluation of potential strategies to slow ash mortality (slam) caused by emerald ash borer (agrilus planipennis): Slam in an urban forest. International Journal of Pest Management 58: 9-23.

McDowall RM (1996) Diadromy and the assembly and restoration of riverine fish communities: A downstream view. Canadian Journal of Fisheries and Aquatic Sciences 53: 219-236.

McHugh N, Thompson S (2011) A rapid ecological network assessment tool and its use in locating habitat extension areas in a changing landscape. Journal for Nature Conservation 19: 236-244.

McLane SC, Aitken SN (2012) Whitebark pine (pinus albicaulis) assisted migration potential: Testing establishment north of the species range. Ecological Applications 22: 142-153.

Meers TL, Enright NJ, Bell TL, Kasel S (2012) Deforestation strongly affects soil seed banks in eucalypt forests: Generalisations in functional traits and implications for restoration. Forest Ecology and Management 266: 94-107.

Mehlman DW (1997) Change in avian abundance across the geographic range in response to environmental change. Ecological Applications 7: 614-624.

Meier ES, Lischke H, Schmatz DR, Zimmermann NE (2012) Climate, competition and connectivity affect future migration and ranges of european trees. Global Ecology and Biogeography 21: 164-178.

Melinchuk R (1995) Ducks unlimiteds landscape approach to habitat conservation. Landscape and Urban Planning 32: 211-217.

Mercader RJ, Siegert NW, McCullough DG (2012) Estimating the influence of population density and dispersal behavior on the ability to detect and monitor agrilus planipennis (coleoptera: Buprestidae) populations. Journal of Economic Entomology 105: 272-281.

Metsoja J-A, Neuenkamp L, Pihu S, Vellak K, Kalwij JM, et al. (2012) Restoration of flooded meadows in estonia - vegetation changes and management indicators. Applied Vegetation Science 15: 231-244.

Meyer J-Y, Fourdrigniez M, Taputuarai R (2012) Restoring habitat for native and endemic plants through the introduction of a fungal pathogen to control the alien invasive tree miconia calvescens in the island of tahiti. Biocontrol 57: 191-198.

Meyer WM, III (2012) Native hawaiian succineids prefer non-native ginger (hedychium spp.) plant species in the kohala mountains, hawaii: Conservation ramifications. American Malacological Bulletin 30: 147-151.

Michelsen A (1992) Mycorrhiza and root nodulation in tree seedlings from 5 nurseries in ethiopia and somalia. Forest Ecology and Management 48: 335-344.

Michelsen A, Lisanework N, Friis I (1993) Impacts of tree plantations in the ethiopian highland on soil fertility, shoot and root-growth, nutrient utilization and mycorrhizal colonization. Forest Ecology and Management 61: 299-324.

Milberg P, Persson TS (1994) Soil seed bank and species recruitment in road verge grassland vegetation. Annales Botanici Fennici 31: 155-162.

Milko LV, Haddad NM, Lance SL (2012) Dispersal via stream corridors structures populations of the endangered st. Francis' satyr butterfly (neonympha mitchellii francisci). Journal of Insect Conservation 16: 263-273.

Millar MA, Byrne M, Nuberg IK, Sedgley M (2012) High levels of genetic contamination in remnant populations of acacia saligna from a genetically divergent planted stand. Restoration Ecology 20: 260-267.

Mills EL, Dermott RM, Roseman EF, Dustin D, Mellina E, et al. (1993) Colonization, ecology, and population-structure of the quagga mussel (bivalvia, dreissenidae) in the lower great-lakes. Canadian Journal of Fisheries and Aquatic Sciences 50: 2305-2314.

Mills JS, Dunham JB, Reeves GH, McMillan JR, Zimmerman CE, et al. (2012) Variability in expression of anadromy by female oncorhynchus mykiss within a river network. Environmental Biology of Fishes 93: 505-517.

Mills M, Pressey RL, Weeks R, Foale S, Ban NC (2010) A mismatch of scales: Challenges in planning for implementation of marine protected areas in the coral triangle. Conservation Letters 3: 291-303.

Mistro DC, Diaz Rodrigues LA, Petrovskii S (2012) Spatiotemporal complexity of biological invasion in a space- and time-discrete predator-prey system with the strong allee effect. Ecological Complexity 9: 16-32.

Mitchley J, Jongepierova I, Fajmon K (2012) Regional seed mixtures for the re-creation of species-rich meadows in the white carpathian mountains: Results of a 10-yr experiment. Applied Vegetation Science 15: 253-263.

Moilanen A (2011) On the limitations of graph-theoretic connectivity in spatial ecology and conservation. Journal of Applied Ecology 48: 1543-1547.

Moir ML, Vesk PA, Brennan KEC, Poulin R, Hughes L, et al. (2012) Considering extinction of dependent species during translocation, ex situ conservation, and assisted migration of threatened hosts. Conservation Biology 26: 199-207.

Molina-Montenegro MA, Cleland EE, Watts SM, Broitman BR (2012) Can a breakdown in competition-colonization tradeoffs help explain the success of exotic species in the california flora? Oikos 121: 389-395.

Monserud RA, Tchebakova NM, Leemans R (1993) Global vegetation change predicted by the modified budyko model. Climatic Change 25: 59-83.

Morgan EH, Richardson CA (2012) Capricious bioinvasions versus uncoordinated management strategies: How the most unlikely invaders can prosper under the current uk legislation framework. Aquatic Conservation-Marine and Freshwater Ecosystems 22: 87-103.

Mossman HL, Brown MJH, Davy AJ, Grant A (2012) Constraints on salt marsh development following managed coastal realignment: Dispersal limitation or environmental tolerance? Restoration Ecology 20: 65-75.

Muller E, Eidesen PB, Ehrich D, Alsos IG (2012) Frequency of local, regional, and long-distance dispersal of diploid and tetraploid saxifraga oppositifolia (saxifragaceae) to arctic glacier forelands. American Journal of Botany 99: 459-471.

Musil CF (1993) Effect of invasive australian acacias on the regeneration, growth and nutrient chemistry of south-african lowland fynbos. Journal of Applied Ecology 30: 361-372.

Nadel H, Frank JH, Knight RJ (1992) Escapees and accomplices - the naturalization of exotic ficus and their associated faunas in florida. Florida Entomologist 75: 29-38.

Neeson TM, Wiley MJ, Adlerstein SA, Riolo RL (2012) How river network structure and habitat availability shape the spatial dynamics of larval sea lampreys. Ecological Modelling 226: 62-70.

Negishi JN, Sagawa S, Sanada S, Kume M, Ohmori T, et al. (2012) Using airborne scanning laser altimetry (lidar) to estimate surface connectivity of floodplain water bodies. River Research and Applications 28: 258-267.

Neri-Arboleda I (2010) Strengths and weaknesses of a population viability analysis for philippine tarsiers (tarsius syrichta). International Journal of Primatology 31: 1192-1207.

Niemela J, Spence JR (1991) Distribution and abundance of an exotic ground-beetle (carabidae) - a test of community impact. Oikos 62: 351-359.

Nilsson C, Brittain JE (1996) Remedial strategies in regulated rivers: Introductory remarks. Regulated Rivers-Research & Management 12: 347-351.

Noatch MR, Suski CD (2012) Non-physical barriers to deter fish movements. Environmental Reviews 20: 71-82.

Northam FE, Old RR, Callihan RH (1993) Little lovegrass (eragrostis-minor) distribution in idaho and washington. Weed Technology 7: 771-775.

Noss RF (1993) A conservation plan for the oregon coast range - some preliminary suggestions. Natural Areas Journal 13: 276-290.

Novak SJ, Mack RN, Soltis PS (1993) Genetic-variation in bromus-tectorum (poaceae) - introduction dynamics in north-america. Canadian Journal of Botany-Revue Canadienne De Botanique 71: 1441-1448.

Nowak CL, Nowak RS, Tausch RJ, Wigand PE (1994) Tree and shrub dynamics in northwestern great-basin woodland and shrub steppe during the late-pleistocene and holocene. American Journal of Botany 81: 265-277.

Nuttle T, Burger LW (1996) Response of breeding bird communities to restoration of hardwood bottomlands; Eversole AG, editor. 228-236 p.

O'Hanley JR (2011) Open rivers: Barrier removal planning and the restoration of free-flowing rivers. Journal of Environmental Management 92: 3112-3120.

Oconnor TG (1991) Local extinction in perennial grasslands - a life-history approach. American Naturalist 137: 753-773.

Odman AM, Martensson L-M, Sjoholm C, Olsson PA (2011) Immediate responses in soil chemistry, vegetation and ground beetles to soil perturbation when implemented as a restoration measure in decalcified sandy grassland. Biodiversity and Conservation 20: 3039-3058.

Ohlemueller R, Huntley B, Normand S, Svenning J-C (2012) Potential source and sink locations for climate-driven species range shifts in europe since the last glacial maximum. Global Ecology and Biogeography 21: 152-163.

Ohtsuka T, Ohsawa M (1994) Accumulation of buried seeds and establishment of ruderal therophytic communities in disturbed habitat, central japan. Vegetatio 110: 83-96.

Olds AD, Connolly RM, Pitt KA, Maxwell PS (2012) Habitat connectivity improves reserve performance. Conservation Letters 5: 56-63.

Olson ER, Doherty JM (2012) The legacy of pipeline installation on the soil and vegetation of southeast wisconsin wetlands. Ecological Engineering 39: 53-62.

Orrock JL, Watling JI (2010) Local community size mediates ecological drift and competition in metacommunities. Proceedings of the Royal Society B-Biological Sciences 277: 2185-2191.

Orth RJ, Luckenbach M, Moore KA (1994) Seed dispersal in a marine macrophyte - implications for colonization and restoration. Ecology 75: 1927-1939.

Orth RJ, Moore KA, Marion SR, Wilcox DJ, Parrish DB (2012) Seed addition facilitates eelgrass recovery in a coastal bay system. Marine Ecology-Progress Series 448: 177-195.

Osborne LL, Bayley PB, Higler LWG, Statzner B, Triska F, et al. (1993) Restoration of lowland streams - an introduction. Freshwater Biology 29: 187-194.

Ostergren J, Nilsson J (2012) Importance of life-history and landscape characteristics for genetic structure and genetic diversity of brown trout (salmo trutta l.). Ecology of Freshwater Fish 21: 119-133.

Overholt WA, Smith JW (1990) Colonization of 6 exotic parasites (hymenoptera) against diatraea-grandiosella (lepidoptera, pyralidae) in corn. Environmental Entomology 19: 1889-1902.

Owers KA, Albanese B, Litts T (2012) Using aerial photography to estimate riparian zone impacts in a rapidly developing river corridor. Environmental Management 49: 543-552.

Pannell J (1997) The maintenance of gynodioecy and androdioecy in a metapopulation. Evolution 51: 10-20.

Parrotta JA (1995) Influence of overstory composition on understory colonization by native species in plantations on a degraded tropical site. Journal of Vegetation Science 6: 627-636.

Pedlar JH, McKenney DW, Beaulieu J, Colombo SJ, McLachlan JS, et al. (2011) The implementation of assisted migration in canadian forests. Forestry Chronicle 87: 766-777.

Perry DA, Borchers JG, Borchers SL, Amaranthus MP (1990) Species migrations and ecosystem stability during climate change - the belowground connection. Conservation Biology 4: 266-274.

Peterson DL, Schreiner EG, Buckingham NM (1997) Gradients, vegetation and climate: Spatial and temporal dynamics in the olympic mountains, USA. Global Ecology and Biogeography Letters 6: 7-17.

Pfeifer M, Passalacqua NG, Bartram S, Schatz B, Croce A, et al. (2010) Conservation priorities differ at opposing species borders of a european orchid. Biological Conservation 143: 2207-2220.

Pfister CA (1998) Extinction, colonization, and species occupancy in tidepool fishes. Oecologia 114: 118-126.

Phillips BL (2012) Range shift promotes the formation of stable range edges. Journal of Biogeography 39: 153-161.

Phillips OL (1997) The changing ecology of tropical forests. Biodiversity and Conservation 6: 291-311.

Pichancourt J-B, Chades I, Firn J, van Klinken RD, Martin TG (2012) Simple rules to contain an invasive species with a complex life cycle and high dispersal capacity. Journal of Applied Ecology 49: 52-62.

Poddubny AG, Galat DL (1995) Habitat associations of upper volga river fishes - effects of reservoirs. Regulated Rivers-Research & Management 11: 67-84.

Poethke HJ, Dytham C, Hovestadt T (2011) A metapopulation paradox: Partial improvement of habitat may reduce metapopulation persistence. American Naturalist 177: 792-799.

Pokluda P, Hauck D, Cizek L (2012) Importance of marginal habitats for grassland diversity: Fallows and overgrown tall-grass steppe as key habitats of endangered ground-beetle carabus hungaricus. Insect Conservation and Diversity 5: 27-36.

Pollard E, Rothery P, Yates TJ (1996) Annual growth rates in newly established populations of the butterfly pararge aegeria. Ecological Entomology 21: 365-369.

Poos MS, Jackson DA (2012) Impact of species-specific dispersal and regional stochasticity on estimates of population viability in stream metapopulations. Landscape Ecology 27: 405-416.

Powell JA (1992) Recent colonization of the san-francisco bay area, california, by exotic moths (lepidoptera, tineoidea, gelechioidea, tortricoidea, pyraloidea). Pan-Pacific Entomologist 68: 105-121.

Pratt JR (1994) Artificial habitats and ecosystem restoration - managing for the future. Bulletin of Marine Science 55: 268-275.

Pratt PD, Center TD (2012) Biocontrol without borders: The unintended spread of introduced weed biological control agents. Biocontrol 57: 319-329.

Primack RB, Miao SL (1992) Dispersal can limit local plant-distribution. Conservation Biology 6: 513-519.

Prospero S, Rigling D (2012) Invasion genetics of the chestnut blight fungus cryphonectria parasitica in switzerland. Phytopathology 102: 73-82.

Puetz S, Groeneveld J, Alves LF, Metzger JP, Huth A (2011) Fragmentation drives tropical forest fragments to early successional states: A modelling study for brazilian atlantic forests. Ecological Modelling 222: 1986-1997.

Pysek P, Prach K (1993) Plant invasions and the role of riparian habitats - a comparison of 4 species alien to central-europe. Journal of Biogeography 20: 413-420.

Ramage BS, Forrestel AB, Moritz MA, O'Hara KL (2012) Sudden oak death disease progression across two forest types and spatial scales. Journal of Vegetation Science 23: 151-163.

Ranius T, Roberge J-M (2011) Effects of intensified forestry on the landscape-scale extinction risk of dead wood dependent species. Biodiversity and Conservation 20: 2867-2882.

Rasmussen R, Hamilton G (2012) An approximate bayesian computation approach for estimating parameters of complex environmental processes in a cellular automata. Environmental Modelling & Software 29: 1-10.

Reed JM (1999) The role of behavior in recent avian extinctions and endangerments. Conservation Biology 13: 232-241.

Regan HM, Syphard AD, Franklin J, Swab RM, Markovchick L, et al. (2012) Evaluation of assisted colonization strategies under global change for a rare, fire-dependent plant. Global Change Biology 18: 936-947.

Reza MIH, Abdullah SA (2011) Regional index of ecological integrity: A need for sustainable management of natural resources. Ecological Indicators 11: 220-229.

Richardson DM, Williams PA, Hobbs RJ (1994) Pine invasions in the southern-hemisphere - determinants of spread and invadability. Journal of Biogeography 21: 511-527.

Riffell SK, Gutzwiller KJ (1996) Plant-species richness in corridor intersections: Is intersection shape influential? Landscape Ecology 11: 157-168.

Risk BB, de Valpine P, Beissinger SR (2011) A robust-design formulation of the incidence function model of metapopulation dynamics applied to two species of rails. Ecology 92: 462-474.

Roberge J-M, Bengtsson SBK, Wulff S, Snall T (2011) Edge creation and tree dieback influence the patch-tracking metapopulation dynamics of a red-listed epiphytic bryophyte. Journal of Applied Ecology 48: 650-658.

Rodriguez-Cabal MA, Stuble KL, Guenard B, Dunn RR, Sanders NJ (2012) Disruption of ant-seed dispersal mutualisms by the invasive asian needle ant (pachycondyla chinensis). Biological Invasions 14: 557-565.

Roger E, Laffan SW, Ramp D (2011) Road impacts a tipping point for wildlife populations in threatened landscapes. Population Ecology 53: 215-227.

Roncal J, Maschinski J, Schaffer B, Gutierrez SM, Walters D (2012) Testing appropriate habitat outside of historic range: The case of amorpha herbacea var. Crenulata (fabaceae). Journal for Nature Conservation 20: 109-116.

Root KV (1998) Evaluating the effects of habitat quality, connectivity, and catastrophes on a threatened species. Ecological Applications 8: 854-865.

Rossong MA, Quijon PA, Snelgrove PVR, Barrett TJ, McKenzie CH, et al. (2012) Regional differences in foraging behaviour of invasive green crab (carcinus maenas) populations in atlantic canada. Biological Invasions 14: 659-669.

Russell EWB, Davis RB, Anderson RS, Rhodes TE, Anderson DS (1993) Recent centuries of vegetational change in the glaciated north-eastern united-states. Journal of Ecology 81: 647-664.

Russellsmith J, Lucas DE (1994) Regeneration of monsoon rain-forest in northern australia - the dormant seed bank. Journal of Vegetation Science 5: 161-168.

Sackett TE, Smith SM, Basiliko N (2012) Exotic earthworm distribution in a mixed-use northern temperate forest region: Influence of disturbance type, development age, and soils. Canadian Journal of Forest Research-Revue Canadienne De Recherche Forestiere 42: 375-381.

Saetersdal M, Birks HJB (1997) A comparative ecological study of norwegian mountain plants in relation to possible future climatic change. Journal of Biogeography 24: 127-152.

Samways MJ (1993) Insects in biodiversity conservation - some perspectives and directives. Biodiversity and Conservation 2: 258-282.

Sato T, Gwo J-C (2011) Demographic and genetic consequences of population subdivision in formosa land-locked salmon oncorhynchus masou formosanus, the southernmost subspecies of the salmonids. Ichthyological Research 58: 209-216.

Saura S, Rubio L (2010) A common currency for the different ways in which patches and links can contribute to habitat availability and connectivity in the landscape. Ecography 33: 523-537.

Sawyer SC, Epps CW, Brashares JS (2011) Placing linkages among fragmented habitats: Do least-cost models reflect how animals use landscapes? Journal of Applied Ecology 48: 668-678.

Schippers P, Verboom J, Vos CC, Jochem R (2011) Metapopulation shift and survival of woodland birds under climate change: Will species be able to track? Ecography 34: 909-919.

Schmiede R, Otte A, Donath TW (2012) Enhancing plant biodiversity in species-poor grassland through plant material transfer - the impact of sward disturbance. Applied Vegetation Science 15: 290-298.

Schuhmacher H, Schillak L (1994) Integrated electrochemical and biogenic deposition of hard material - a nature-like colonization substrate. Bulletin of Marine Science 55: 672-679.

Schwartz MW (1992) Potential effects of global climate change on the biodiversity of plants. Forestry Chronicle 68: 462-471.

Schwinning S, Parsons AJ (1996) A spatially explicit population model of stoloniferous n-fixing legumes in mixed pasture with grass. Journal of Ecology 84: 815-826.

Scott AJ, Morgan JW (2012) Recovery of soil and vegetation in semi-arid australian old fields. Journal of Arid Environments 76: 61-71.

Scribner KT, Stuwe M (1994) Genetic-relationships among alpine ibex capra-ibex populations reestablished from a common ancestral source. Biological Conservation 69: 137-143.

Seuffert ME, Martin PR (2012) A lentic dweller in lotic habitats: The behavior of the invasive south american apple snail pomacea canaliculata in flowing water. Aquatic Ecology 46: 129-142.

Sheehy J, Taylor CM, Norris DR (2011) The importance of stopover habitat for developing effective conservation strategies for migratory animals. Journal of Ornithology 152: 161-168.

Shier DM, Swaisgood RR (2012) Fitness costs of neighborhood disruption in translocations of a solitary mammal. Conservation Biology 26: 116-123.

Shirk AJ, Cushman SA (2011) Sgd: Software for estimating spatially explicit indices of genetic diversity. Molecular Ecology Resources 11: 922-934.

Simberloff D (2012) Risks of biological control for conservation purposes. Biocontrol 57: 263-276.

Sirami C, Monadjem A (2012) Changes in bird communities in swaziland savannas between 1998 and 2008 owing to shrub encroachment. Diversity and Distributions 18: 390-400.

Skelly DK, Meir E (1997) Rule-based models for evaluating mechanisms of distributional change. Conservation Biology 11: 531-538.

Skelsey P, Rossing WAH, Kessel GJT, van der Werf W (2010) Invasion of phytophthora infestans at the landscape level: How do spatial scale and weather modulate the consequences of spatial heterogeneity in host resistance? Phytopathology 100: 1146-1161.

Smallwood KS (1994) Site invasibility by exotic birds and mammals. Biological Conservation 69: 251-259.

Smith RIL (1991) Exotic sporomorpha as indicators of potential immigrant colonists in antarctica. Grana 30: 313-324.

Snep RPH, WallisDeVries MF, Opdam P (2011) Conservation where people work: A role for business districts and industrial areas in enhancing endangered butterfly populations? Landscape and Urban Planning 103: 94-101.

Solomon AM, Kirilenko AP (1997) Climate change and terrestrial biomass: What if trees do not migrate! Global Ecology and Biogeography Letters 6: 139-148.

Spencer W, Rustigian-Romsos H, Strittholt J, Scheller R, Zielinski W, et al. (2011) Using occupancy and population models to assess habitat conservation opportunities for an isolated carnivore population. Biological Conservation 144: 788-803.

Spromberg JA, John BM, Landis WG (1998) Metapopulation dynamics: Indirect effects and multiple distinct outcomes in ecological risk assessment. Environmental Toxicology and Chemistry 17: 1640-1649.

Stanford JA, Ward JV, Liss WJ, Frissell CA, Williams RN, et al. (1996) A general protocol for restoration of regulated rivers. Regulated Rivers-Research & Management 12: 391-413.

Stasko AD, Patenaude T, Strecker AL, Arnott SE (2012) Portage connectivity does not predict establishment success of canoe-mediated dispersal for crustacean zooplankton. Aquatic Ecology 46: 9-24.

Steinger T, Korner C, Schmid B (1996) Long-term persistence in a changing climate: DNA analysis suggests very old ages of clones of alpine carex curvula. Oecologia 105: 94-99.

Stelter C, Reich M, Grimm V, Wissel C (1997) Modelling persistence in dynamic landscapes: Lessons from a metapopulation of the grasshopper bryodema tuberculata. Journal of Animal Ecology 66: 508-518.

Stevenson MJ, Bullock JM, Ward LK (1995) Re-creating semi-natural communities: Effect of sowing rate on establishment of calcareous grassland. Restoration Ecology 3: 279-289.

Stewart CW, van der Ree R (2010) A voronoi diagram based population model for social species of wildlife. Ecological Modelling 221: 1554-1568.

Stoddard ST (2010) Continuous versus binary representations of landscape heterogeneity in spatially-explicit models of mobile populations. Ecological Modelling 221: 2409-2414.

Stoker RL, Ferris DK, Grant WE, Folse LJ (1994) Simulating colonization by exotic species - a model of the red imported fire ant (solenopsis-invicta) in north-america. Ecological Modelling 73: 281-292.

Strykstra RJ, Bekker RM, Verweij GL (1996) Establishment of rhinanthus angustifolius in a successional hayfield after seed dispersal by mowing machinery. Acta Botanica Neerlandica 45: 557-562.

Sutor A, Schwarz S (2012) Home ranges of raccoon dogs (nyctereutes procyonoides, gray, 1834) in southern brandenburg, germany. European Journal of Wildlife Research 58: 85-97.

Swanson BJ, Johnson DR (1996) Spatial and temporal trends and effects of population size on the frequency of color phenotypes in the wild red fox (vulpes vulpes). Canadian Journal of Zoology-Revue Canadienne De Zoologie 74: 1622-1631.

Sweitzer RA, Jenkins SH, Berger J (1997) Near-extinction of porcupines by mountain lions and consequences of ecosystem change in the great basin desert. Conservation Biology 11: 1407-1417.

Sykes MT, Prentice IC (1996) Climate change, tree species distributions and forest dynamics: A case study in the mixed conifer northern hardwoods zone of northern europe. Climatic Change 34: 161-177.

Taillefer AG, Wheeler TA (2012) Community assembly of diptera following restoration of mined boreal bogs: Taxonomic and functional diversity. Journal of Insect Conservation 16: 165-176.

Tamo M, Baumgartner J, Delucchi V, Herren HR (1993) Assessment of key factors responsible for the pest status of the bean flower thrips megalurothrips-sjostedti (thysanoptera, thripidae) in west-africa. Bulletin of Entomological Research 83: 251-258.

Taylor AC, Walker FM, Goldingay RL, Ball T, van der Ree R (2011) Degree of landscape fragmentation influences genetic isolation among populations of a gliding mammal. PloS one 6: e26651.

Taylor J, Paine C, Fitzgibbon J (1995) From greenbelt to greenways - 4 canadian case-studies. Landscape and Urban Planning 33: 47-64.

Tegner MJ, Dayton PK (1991) Sea-urchins, el-ninos, and the long-term stability of southern california kelp forest communities. Marine Ecology-Progress Series 77: 49-63.

Teketay D, Granstrom A (1995) Soil seed banks in dry afromontane forests of ethiopia. Journal of Vegetation Science 6: 777-786.

Tian Y, Wu J, Smith AT, Wang T, Kou X, et al. (2011) Population viability of the siberian tiger in a changing landscape: Going, going and gone? Ecological Modelling 222: 3166-3180.

Tilman D, Lehman CL, Yin CJ (1997) Habitat destruction, dispersal, and deterministic extinction in competitive communities. American Naturalist 149: 407-435.

Towns DR, Daugherty CH (1994) Patterns of range contractions and extinctions in the new-zealand herpetofauna following human colonization. New Zealand Journal of Zoology 21: 325-339.

Tribe GD (1991) Phenology of pinus-radiata log colonization by the red-haired pine bark beetle hylurgus-ligniperda (fabricius) (coleoptera, scolytidae) in the south-western cape province. Journal of the Entomological Society of Southern Africa 54: 1-7.

Tribe GD (1992) Colonization sites on pinus-radiata logs of the bark beetles, orthotomicus-erosus, hylastes-angustatus and hylurgus-ligniperda (coleoptera, scolytidae). Journal of the Entomological Society of Southern Africa 55: 77-84.

Trisurat Y, Pattanavibool A, Gale GA, Reed DH (2010) Improving the viability of large-mammal populations by using habitat and landscape models to focus conservation planning. Wildlife Research 37: 401-412.

Tsai J-S, Venne LS, McMurry ST, Smith LM (2012) Local and landscape influences on plant communities in playa wetlands. Journal of Applied Ecology 49: 174-181.

Tsuji M, Ushimaru A, Osawa T, Mitsuhashi H (2011) Paddy-associated frog declines via urbanization: A test of the dispersal-dependent-decline hypothesis. Landscape and Urban Planning 103: 318-325.

Tucker JK (1994) Colonization of unionid bivalves by the zebra mussel, dreissena-polymorpha, in pool-26 of the mississippi river. Journal of Freshwater Ecology 9: 129-134.

Turner MG, Romme WH, Gardner RH, Hargrove WW (1997) Effects of fire size and pattern on early succession in yellowstone national park. Ecological Monographs 67: 411-433.

Tyser RW, Worley CA (1992) Alien flora in grasslands adjacent to road and trail corridors in glacier national-park, montana (USA). Conservation Biology 6: 253-262.

Um JS, Wright R (1996) Pipeline construction and reinstatement monitoring: Current practice, limitations and the value of airborne videography. Science of the Total Environment 186: 221-230.

Unghire JM, Sutton-Grier AE, Flanagan NE, Richardson CJ (2011) Spatial impacts of stream and wetland restoration on riparian soil properties in the north carolina piedmont. Restoration Ecology 19: 738-746.

Vaclavik T, Meentemeyer RK (2012) Equilibrium or not? Modelling potential distribution of invasive species in different stages of invasion. Diversity and Distributions 18: 73-83.

Van Apeldoorn RC, Knaapen JP, Schippers P, Verboom J, Van Engen H, et al. (1998) Applying ecological knowledge in landscape planning: A simulation model as a tool to evaluate scenarios for the badger in the netherlands. Landscape and Urban Planning 41: 57-69.

van Langevelde F, Schotman A, Claassen F, Sparenburg G (2000) Competing land use in the reserve site selection problem. Landscape Ecology 15: 243-256.

van Noordwijk CGE, Boer P, Mabelis AA, Verberk WCEP, Siepel H (2012) Life-history strategies as a tool to identify conservation constraints: A case-study on ants in chalk grasslands. Ecological Indicators 13: 303-313.

vanDorp D, Schippers P, vanGroenendael JM (1997) Migration rates of grassland plants along corridors in fragmented landscapes assessed with a cellular automation model. Landscape Ecology 12: 39-50.

Vargas P, Heleno R, Traveset A, Nogales M (2012) Colonization of the galapagos islands by plants with no specific syndromes for long-distance dispersal: A new perspective. Ecography 35: 33-43.

Veale AJ, Clout MN, Gleeson DM (2012) Genetic population assignment reveals a long-distance incursion to an island by a stoat (mustela erminea). Biological Invasions 14: 735-742.

Veit RR, Pyle P, McGowan JA (1996) Ocean warming and long-term change in pelagic bird abundance within the california current system. Marine Ecology-Progress Series 139: 11-18.

Verburg PH, Koomen E, Hilferink M, Perez-Soba M, Lesschen JP (2012) An assessment of the impact of climate adaptation measures to reduce flood risk on ecosystem services. Landscape Ecology 27: 473-486.

Villard MA, Merriam G, Maurer BA (1995) Dynamics in subdivided populations of neotropical migratory birds in a fragmented temperate forest. Ecology 76: 27-40.

Visconti P, Pressey RL, Segan DB, Wintle BA (2010) Conservation planning with dynamic threats: The role of spatial design and priority setting for species' persistence. Biological Conservation 143: 756-767.

Vora RS (1994) Integrating old-growth forest into managed landscapes - a northern great-lakes perspective. Natural Areas Journal 14: 113-123.

Vucetich JA, Creel S (1999) Ecological interactions, social organization, and extinction risk in african wild dogs. Conservation Biology 13: 1172-1182.

Wardle P, Coleman MC (1992) Evidence for rising upper limits of 4 native new-zealand forest trees. New Zealand Journal of Botany 30: 303-314.

Ware C, Bergstrom DM, Muller E, Alsos IG (2012) Humans introduce viable seeds to the arctic on footwear. Biological Invasions 14: 567-577.

Wasserman TN, Cushman SA, Shirk AS, Landguth EL, Littell JS (2012) Simulating the effects of climate change on population connectivity of american marten (martes americana) in the northern rocky mountains, USA. Landscape Ecology 27: 211-225.

Watts K, Eycott AE, Handley P, Ray D, Humphrey JW, et al. (2010) Targeting and evaluating biodiversity conservation action within fragmented landscapes: An approach based on generic focal species and least-cost networks. Landscape Ecology 25: 1305-1318.

Webb JK, Shine R (1997) A field study of spatial ecology and movements of a threatened snake species, hoplocephalus bungaroides. Biological Conservation 82: 203-217.

Webber BL, Scott JK (2012) Rapid global change: Implications for defining natives and aliens. Global Ecology and Biogeography 21: 305-311.

Weber TC, Allen WL (2010) Beyond on-site mitigation: An integrated, multi-scale approach to environmental mitigation and stewardship for transportation projects. Landscape and Urban Planning 96: 240-256.

Welker JM, Molau U, Parsons AN, Robinson CH, Wookey PA (1997) Responses of dryas octopetala to itex environmental manipulations: A synthesis with circumpolar comparisons. Global Change Biology 3: 61-73.

Wennergren U, Ruckelshaus M, Kareiva P (1995) The promise and limitations of spatial models in conservation biology. Oikos 74: 349-356.

Westbrook JK, Eyster RS, Allen CT (2011) A model for long-distance dispersal of boll weevils (coleoptera: Curculionidae). International Journal of Biometeorology 55: 585-593.

Whited D, Galatowitsch S, Tester JR, Schik K, Lehtinen R, et al. (2000) The importance of local and regional factors in predicting effective conservation - planning strategies for wetland bird communities in agricultural and urban landscapes. Landscape and Urban Planning 49: 49-65.

Whitehead AL, Elliott GP, McIntosh AR (2010) Large-scale predator control increases population viability of a rare new zealand riverine duck. Austral Ecology 35: 722-730.

Whittaker RJ, Jones SH (1994) Structure in re-building insular ecosystems - an empirically derived model. Oikos 69: 524-530.

Wijayratne UC, Pyke DA (2012) Burial increases seed longevity of two artemisia tridentata (asteraceae) subspecies. American Journal of Botany 99: 438-447.

Wilkie DS, Curran B, Tshombe R, Morelli GA (1998) Modeling the sustainability of subsistence farming and hunting in the ituri forest of zaire. Conservation Biology 12: 137-147.

Williams LR, Echelle AA, Toepfer CS, Williams MG, Fisher WL (1999) Simulation modeling of population viability for the leopard darter (percidae : Percina pantherina). Southwestern Naturalist 44: 470-477.

Wilson JB, Rapson GL, Sykes MT, Watkins AJ, Williams PA (1992) Distributions and climatic correlations of some exotic species along roadsides in south island, new-zealand. Journal of Biogeography 19: 183-193.

Wilson RJ, Davies ZG, Thomas CD (2010) Linking habitat use to range expansion rates in fragmented landscapes: A metapopulation approach. Ecography 33: 73-82.

Wood PA, Samways MJ (1991) Landscape element pattern and continuity of butterfly flight paths in an ecologically landscaped botanical garden, natal, south-africa. Biological Conservation 58: 149-166.

Woodcock BA, McDonald AW, Pywell RF (2011) Can long-term floodplain meadow recreation replicate species composition and functional characteristics of target grasslands? Journal of Applied Ecology 48: 1070-1078.

Woodcock BA, Westbury DB, Brook AJ, Lawson CS, Edwards AR, et al. (2012) Effects of seed addition on beetle assemblages during the re-creation of species-rich lowland hay meadows. Insect Conservation and Diversity 5: 19-26.

Wookey PA, Parsons AN, Welker JM, Potter JA, Callaghan TV, et al. (1993) Comparative responses of phenology and reproductive development to simulated environmental-change in sub-arctic and high arctic plants. Oikos 67: 490-502.

Wookey PA, Robinson CH, Parsons AN, Welker JM, Press MC, et al. (1995) Environmental constraints on the growth, photosynthesis and reproductive development of dryas-octopetala at a high arctic polar semidesert, svalbard. Oecologia 102: 478-489.

Wu C-F, Lin Y-P, Lin S-H (2011) A hybrid scheme for comparing the effects of bird diversity conservation approaches on landscape patterns and biodiversity in the shangan sub-watershed in taiwan. Journal of Environmental Management 92: 1809-1820.

Wu JG, Levin SA (1994) A spatial patch dynamic modeling approach to pattern and process in an annual grassland. Ecological Monographs 64: 447-464.

Yahner TG, Korostoff N, Johnson TP, Battaglia AM, Jones DR (1995) Cultural landscapes and landscape ecology in contemporary greenway planning, design and management - a case-study. Landscape and Urban Planning 33: 295-316.

Yang C-C, Ascunce MS, Luo L-Z, Shao J-G, Shih C-J, et al. (2012) Propagule pressure and colony social organization are associated with the successful invasion and rapid range expansion of fire ants in china. Molecular Ecology 21: 817-833.

Yaussy DA, Sutherland EK, Hale BJ (1996) Rule-based, individual-tree regeneration model for forest simulators; Skovsgaard JPJVK, editor. 176-182 p.

Yeates AG, Schooler SS, Garono RJ, Buckley YM (2012) Biological control as an invasion process: Disturbance and propagule pressure affect the invasion success of lythrum salicaria biological control agents. Biological Invasions 14: 255-271.

Yu D, Jiang Y, Kang M, Tian Y, Duan J (2011) Integrated urban land-use planning based on improving ecosystem service: Panyu case, in a typical developed area of china. Journal of Urban Planning and Development-Asce 137: 448-458.

Zehfuss KP, Hightower JE, Pollock KH (1999) Abundance of gulf sturgeon in the apalachicola river, florida. Transactions of the American Fisheries Society 128: 130-143.

Zeller KA, Nijhawan S, Salom-Perez R, Potosme SH, Hines JE (2011) Integrating occupancy modeling and interview data for corridor identification: A case study for jaguars in nicaragua. Biological Conservation 144: 892-901.

Zhu K, Woodall CW, Clark JS (2012) Failure to migrate: Lack of tree range expansion in response to climate change. Global Change Biology 18: 1042-1052.

Ziv Y (1998) The effect of habitat heterogeneity on species diversity patterns: A community-level approach using an object-oriented landscape simulation model (shalom). Ecological Modelling 111: 135-170.
