# Supplementary material for: The Trajectory of Dispersal Research in Conservation Biology. Systematic Review
Source: PLoS One. 2014 Apr 17;9(4):e95053. doi: 10.1371/journal.pone.0095053 (PMC3990620; doi:10.1371/journal.pone.0095053)
Supplement: Appendix S2 — Risk of bias from special issues, commonly studied species or prolific authors (DOCX) [file pone.0095053.s002.docx]

**Appendix S2. Little risk of bias.**

We examined the risk that bias may be introduced by special editions, repeated studies on the same species or by multiple publications from the same author. Our sampling included some related papers from special editions (six papers out of 52 in the invasives topic from the recent time period, 5/53 from recent restoration papers, and 4/18 from land planning papers from the old time period). The potential for these related papers to bias results is negligible, both because they represent a small proportion of the total number of papers, and because the topics they canvassed were broad and not directly related to dispersal (Role of biocontrol in wildlands, from Biocontrol 14 (3) 2012; Vegetation restoration, from Applied Vegetation Science 15 (2) 2012; greenways from Landscape and Urban Planning 33 (1-3) 1995).

Of the 228 papers that focussed on a single genus or species, one genus (*Ficus*) appeared in three papers, and two species appeared in two papers (*Zostera marina* and *Saxifraga oppositifolia*), indicating the database was not biased by repeated studies on the same species. Similarly, the database was not biased by repeated studies by the same author because individual authors contributed to few papers. Of the 1588 authors on reviewed papers, twenty three were named on three papers, and two authors were named on five papers. The rest were named on either one or two papers.
