# Supplementary material for: The Trajectory of Dispersal Research in Conservation Biology. Systematic Review
Source: PLoS One. 2014 Apr 17;9(4):e95053. doi: 10.1371/journal.pone.0095053 (PMC3990620; doi:10.1371/journal.pone.0095053)
Supplement: Table S1 — A description of the eighteen response variables that formed the basis of our review. (DOCX) [file pone.0095053.s003.docx]

Table S1. Eighteen response variables formed the basis of our review. Most were categorical responses (see Table 2).

| **Question** | **Response** | **Description** |
| --- | --- | --- |
| Summary statistics about the data base | Importance of dispersal in paper | Identify the extent to which dispersal is used in the paper; three categories in decreasing order of importance: aim/main focus of paper; used in analysis (ie: values for dispersal used in statistical or simulation modelling); used in interpretation (in discussion). If dispersal was a main aim, it was also used in analysis and used in the discussion. |
|  | Source | The source of the dispersal data used in the paper under review. If the paper under review and additional sources were used, we reported data in the paper under review. If multiple references were cited, we used titles to select the paper that most related to dispersal. Where multiple titles were equally relevant, the first cited paper was used. If the source did not provide dispersal data but referred to other work, we did not collect data that required a source for that review paper (Questions 2 and 3). |
|  | Study type | The type of paper: modelling, empirical, review. |
| Question 1 | Use of dispersal knowledge | What is the problem being solved by the paper that needed dispersal information? The aim of this question was to assess the number and nature of applications to which dispersal information is put within each of the five topics. |
| Question 2 | Method | The method used to collect dispersal data. Method data comes from the 'source' (see below). If multiple methods were used, we reported the first method cited. 'Modelling' was the method where dispersal information was an output of a model. Where a paper used modelling that incorporated dispersal data, the method referred to how the data were collected. |
| Question 3 | Relevance | A score of 1 (source uses a different species in a different environment); 2 (same species in a different environment, or different species in the same environment), or; 3 (same species, same environment). If the paper being reviewed was also the source then relevance = 3. |
|  | Dispersal statistic | Categories were aimed at representing the quality of the dispersal statistic. In that context, a dispersal distribution (such as a dispersal kernel) represents high quality dispersal data, requiring high quality field data to obtain. A single value of dispersal, such as the maximum dispersal distance, is a much simpler and lower quality metric. The category 'inferred-dispersal-in-categories' refers to studies where taxa were categorised as, for example, strong versus weak dispersers, based on morphology. 'Inferred dispersal from occupancy' occurred when qualitative inference about dispersal was drawn from occupancy data. This was highly, but not perfectly, correlated with "habitat occupancy" in Method because some habitat occupancy methods estimated dispersal distributions. |
|  | Sample size | Sample size is method specific. For example, number recaptured, number radio-tracked, number of sites (for community level work and habitat occupancy), number of individuals x number of loci (for genetics). |
|  | Study duration | Number of years over which data were collected. |
|  | Age of source | Year of reviewed paper minus year of source (= 0 when the review paper is the source) |
| Question 4 | Non-dispersal knowledge gap | A count of the number of non-dispersal knowledge gaps highlighted in the paper. This helps to put dispersal knowledge gaps in context: how prevalent are dispersal knowledge gaps compared with other gaps? |
|  | Dispersal knowledge gap | Was dispersal information identified as a knowledge gap? |
|  | Kind of dispersal knowledge gap | Knowledge gaps related to dispersal were classified into categories based on the specific aspect of dispersal that authors reported as needing additional research. |
|  | Use of dispersal knowledge | What is the problem being solved by the paper that needed dispersal information? The aim of this question was to assess the number and nature of applications to which dispersal information is put within each of the five topics. |
| Question 5 | Consequences for study if dispersal data not available | How would the study conclusions be changed without dispersal data? For a study that had the aim of measuring dispersal, without dispersal data, the study would not be possible. At the other extreme, a modelling study that made up its dispersal parameters would be unaffected by the absence of dispersal data. |
|  | Consequences for biodiversity if dispersal data not available | What are the broader implications of not having dispersal information? This may have been highlighted by the author in the discussion, but was most often up the reviewer to draw an appropriate inference, guided by the broad categories. |
| General summary statistics | Taxon | Broad classification of the organisms studied. |
|  | Biome | Freshwater, marine, terrestrial. Amphibians were classified as terrestrial, studies in salt-marshes and mangroves as marine. Some studies included more than one biome, in which case both were counted. |
|  | Region | Defines broad global regions. |
