# Supplementary material for: The Trajectory of Dispersal Research in Conservation Biology. Systematic Review
Source: PLoS One. 2014 Apr 17;9(4):e95053. doi: 10.1371/journal.pone.0095053 (PMC3990620; doi:10.1371/journal.pone.0095053)
Supplement: Table S2 — Test statistics from all analyses. (DOCX) [file pone.0095053.s004.docx]

Table S2. Wald's test statistic and P values for each of the generalised linear mixed models.

| Response | Category | Factor | Wald | P |
| --- | --- | --- | --- | --- |
| Study type | Empirical | age1 | 0.50 | 0.615 |
| Study type | Empirical | topic | 44.80 | 0.000 |
| Study type | Empirical | topic:age | 14.84 | 0.005 |
| Study type | Model including some empirical data | age1 | 0.23 | 0.819 |
| Study type | Model including some empirical data | topic | 32.43 | 0.000 |
| Study type | Model including some empirical data | topic:age | 3.97 | 0.410 |
| Study type | Model or theory no empirical data | age1 | 0.00 | 0.996 |
| Study type | Model or theory no empirical data | topic | 16.22 | 0.003 |
| Study type | Model or theory no empirical data | topic:age | 3.03 | 0.553 |
| Study type | Review | age1 | -0.74 | 0.461 |
| Study type | Review | topic | 17.81 | 0.001 |
| Study type | Review | topic:age | 7.57 | 0.108 |
| Method | Habitat occupancy | age1 | -0.26 | 0.796 |
| Method | Habitat occupancy | topic | 12.82 | 0.012 |
| Method | Habitat occupancy | topic:age | 6.70 | 0.152 |
| Method | Expert opinion | age1 | -0.01 | 0.990 |
| Method | Expert opinion | topic | 4.58 | 0.334 |
| Method | Expert opinion | topic:age | 0.50 | 0.973 |
| Method | Modelling | age1 | -0.57 | 0.569 |
| Method | Modelling | topic | 6.05 | 0.195 |
| Method | Modelling | topic:age | 2.21 | 0.698 |
| Method | Direct observation | age1 | 0.00 | 0.997 |
| Method | Direct observation | topic | 1.07 | 0.900 |
| Method | Direct observation | topic:age | 0.25 | 0.993 |
| Method | Genetics | age1 | 2.34 | 0.019 |
| Method | Genetics | topic | 0.45 | 0.978 |
| Method | Genetics | topic:age | 1.28 | 0.864 |
| Method | Review | age1 | 0.46 | 0.649 |
| Method | Review | topic | 0.93 | 0.920 |
| Method | Review | topic:age | 1.96 | 0.743 |
| Method | Mark-recapture | age1 | 0.57 | 0.567 |
| Method | Mark-recapture | topic | 8.35 | 0.079 |
| Method | Mark-recapture | topic:age | 1.44 | 0.837 |
| Method | Measure arrival from known sources | age1 | -0.01 | 0.995 |
| Method | Measure arrival from known sources | topic | 3.25 | 0.517 |
| Method | Measure arrival from known sources | topic:age | 2.11 | 0.715 |
| Method | Theoretical | age1 | -1.03 | 0.303 |
| Method | Theoretical | topic | 0.01 | 1.000 |
| Method | Theoretical | topic:age | 0.84 | 0.933 |
| Method | Radio-tracking | age1 | 0.00 | 0.997 |
| Method | Radio-tracking | topic | 2.17 | 0.705 |
| Method | Radio-tracking | topic:age | 2.41 | 0.661 |
| Source | Current paper | age1 | -0.02 | 0.988 |
| Source | Current paper | topic | 2.74 | 0.602 |
| Source | Current paper | topic:age | 1.40 | 0.844 |
| Source | Other paper | age1 | 0.44 | 0.660 |
| Source | Other paper | topic | 1.70 | 0.791 |
| Source | Other paper | topic:age | 1.36 | 0.852 |
| Relevance of source paper | Same species same environment | age1 | -0.66 | 0.510 |
| Relevance of source paper | Same species same environment | topic | 8.44 | 0.077 |
| Relevance of source paper | Same species same environment | topic:age | 8.96 | 0.062 |
| Dispersal statistic | Dispersal distribution | age1 | 1.46 | 0.144 |
| Dispersal statistic | Dispersal distribution | topic | 8.72 | 0.069 |
| Dispersal statistic | Dispersal distribution | topic:age | 2.64 | 0.620 |
| Dispersal statistic | Dispersal single value | age1 | -0.26 | 0.799 |
| Dispersal statistic | Dispersal single value | topic | 1.91 | 0.751 |
| Dispersal statistic | Dispersal single value | topic:age | 1.99 | 0.737 |
| Dispersal statistic | Genetics inferred dispersal | age1 | 1.40 | 0.160 |
| Dispersal statistic | Genetics inferred dispersal | topic | 0.05 | 1.000 |
| Dispersal statistic | Genetics inferred dispersal | topic:age | 0.14 | 0.998 |
| Dispersal statistic | Inferred dispersal from occupancy data | age1 | -1.52 | 0.128 |
| Dispersal statistic | Inferred dispersal from occupancy data | topic | 22.92 | 0.000 |
| Dispersal statistic | Inferred dispersal from occupancy data | topic:age | 2.07 | 0.722 |
| Dispersal statistic | Inferred dispersal in categories | age1 | -0.97 | 0.331 |
| Dispersal statistic | Inferred dispersal in categories | topic | 1.03 | 0.905 |
| Dispersal statistic | Inferred dispersal in categories | topic:age | 3.87 | 0.423 |
| Dispersal statistic | Number/proportion of individuals that migrate/disperse | age1 | 0.01 | 0.996 |
| Dispersal statistic | Number/proportion of individuals that migrate/disperse | topic | 6.57 | 0.160 |
| Dispersal statistic | Number/proportion of individuals that migrate/disperse | topic:age | 3.14 | 0.535 |
| Non-dispersal knowledge gap | Non-dispersal gaps identified | age1 | -0.62 | 0.532 |
| Non-dispersal knowledge gap | Non-dispersal gaps identified | topic | 6.53 | 0.163 |
| Non-dispersal knowledge gap | Non-dispersal gaps identified | topic:age | 10.43 | 0.034 |
| Dispersal knowledge gap | Dispersal identified as a knowledge gap | age1 | 1.10 | 0.270 |
| Dispersal knowledge gap | Dispersal identified as a knowledge gap | topic | 17.06 | 0.002 |
| Dispersal knowledge gap | Dispersal identified as a knowledge gap | topic:age | 5.24 | 0.263 |
| Consequences for study if dispersal data not available | Conclusions / interpretation from study weakened / unreliable | age1 | -0.81 | 0.415 |
| Consequences for study if dispersal data not available | Conclusions / interpretation from study weakened / unreliable | topic | 2.29 | 0.683 |
| Consequences for study if dispersal data not available | Conclusions / interpretation from study weakened / unreliable | topic:age | 0.85 | 0.932 |
| Consequences for study if dispersal data not available | Makes no difference | age1 | -0.02 | 0.982 |
| Consequences for study if dispersal data not available | Makes no difference | topic | 3.34 | 0.503 |
| Consequences for study if dispersal data not available | Makes no difference | topic:age | 0.18 | 0.996 |
| Consequences for study if dispersal data not available | Part of study not possible | age1 | 0.29 | 0.768 |
| Consequences for study if dispersal data not available | Part of study not possible | topic | 0.24 | 0.993 |
| Consequences for study if dispersal data not available | Part of study not possible | topic:age | 0.80 | 0.939 |
| Consequences for study if dispersal data not available | Study not possible | age1 | 0.89 | 0.371 |
| Consequences for study if dispersal data not available | Study not possible | topic | 9.82 | 0.044 |
| Consequences for study if dispersal data not available | Study not possible | topic:age | 6.29 | 0.179 |
| Consequences for biodiversity if dispersal data not available | Cannot determine effectiveness of management actions | age1 | 0.01 | 0.996 |
| Consequences for biodiversity if dispersal data not available | Cannot determine effectiveness of management actions | topic | 46.63 | 0.000 |
| Consequences for biodiversity if dispersal data not available | Cannot determine effectiveness of management actions | topic:age | 8.39 | 0.078 |
| Consequences for biodiversity if dispersal data not available | Cannot model/predict extinction risk | age1 | 0.00 | 0.997 |
| Consequences for biodiversity if dispersal data not available | Cannot model/predict extinction risk | topic | 10.10 | 0.039 |
| Consequences for biodiversity if dispersal data not available | Cannot model/predict extinction risk | topic:age | 0.00 | 1.000 |
| Consequences for biodiversity if dispersal data not available | Cannot predict ecological processes | age1 | 1.15 | 0.249 |
| Consequences for biodiversity if dispersal data not available | Cannot predict ecological processes | topic | 22.90 | 0.000 |
| Consequences for biodiversity if dispersal data not available | Cannot predict ecological processes | topic:age | 7.68 | 0.104 |
| Consequences for biodiversity if dispersal data not available | Cannot predict effects of, or adaptation to, climate change | age1 | -0.20 | 0.841 |
| Consequences for biodiversity if dispersal data not available | Cannot predict effects of, or adaptation to, climate change | topic | 0.00 | 1.000 |
| Consequences for biodiversity if dispersal data not available | Cannot predict effects of, or adaptation to, climate change | topic:age | 0.00 | 1.000 |
| Consequences for biodiversity if dispersal data not available | None | age1 | -1.63 | 0.104 |
| Consequences for biodiversity if dispersal data not available | None | topic | 3.41 | 0.492 |
| Consequences for biodiversity if dispersal data not available | None | topic:age | 0.90 | 0.925 |
| Taxon | Mammal | age1 | 0.46 | 0.649 |
| Taxon | Mammal | topic | 4.66 | 0.325 |
| Taxon | Mammal | topic:age | 1.66 | 0.798 |
| Taxon | Bird | age1 | 0.69 | 0.493 |
| Taxon | Bird | topic | 4.65 | 0.325 |
| Taxon | Bird | topic:age | 1.46 | 0.833 |
| Taxon | Fish | age1 | -0.46 | 0.649 |
| Taxon | Fish | topic | 0.17 | 0.996 |
| Taxon | Fish | topic:age | 1.34 | 0.855 |
| Taxon | Plant | age1 | -3.00 | 0.003 |
| Taxon | Plant | topic | 32.19 | 0.000 |
| Taxon | Plant | topic:age | 7.21 | 0.125 |
| Taxon | Insect | age1 | 0.00 | 0.999 |
| Taxon | Insect | topic | 11.21 | 0.024 |
| Taxon | Insect | topic:age | 0.79 | 0.940 |
| Taxon | Non-insect invertebrate | age1 | -0.97 | 0.333 |
| Taxon | Non-insect invertebrate | topic | 2.33 | 0.675 |
| Taxon | Non-insect invertebrate | topic:age | 2.34 | 0.673 |
| Taxon | Ecosystem | age1 | 0.00 | 0.997 |
| Taxon | Ecosystem | topic | 0.03 | 1.000 |
| Taxon | Ecosystem | topic:age | 0.80 | 0.939 |
| Taxon | Vertebrates | age1 | 0.76 | 0.450 |
| Taxon | Vertebrates | topic | 9.64 | 0.047 |
| Taxon | Vertebrates | topic:age | 0.65 | 0.958 |
| Taxon | Invertebrates | age1 | -0.73 | 0.465 |
| Taxon | Invertebrates | topic | 13.13 | 0.011 |
| Taxon | Invertebrates | topic:age | 2.27 | 0.686 |
| Biome | Terrestrial | age1 | 0.17 | 0.864 |
| Biome | Terrestrial | topic | 4.29 | 0.368 |
| Biome | Terrestrial | topic:age | 8.97 | 0.062 |
| Biome | Marine | age1 | -0.25 | 0.805 |
| Biome | Marine | topic | 1.84 | 0.764 |
| Biome | Marine | topic:age | 4.41 | 0.354 |
| Biome | Freshwater | age1 | 0.93 | 0.354 |
| Biome | Freshwater | topic | 6.45 | 0.168 |
| Biome | Freshwater | topic:age | 5.71 | 0.222 |
| Region | Global | age1 | -0.63 | 0.527 |
| Region | Global | topic | 6.36 | 0.174 |
| Region | Global | topic:age | 3.37 | 0.498 |
| Region | Africa | age1 | 0.08 | 0.939 |
| Region | Africa | topic | 12.25 | 0.016 |
| Region | Africa | topic:age | 1.08 | 0.898 |
| Region | Europe | age1 | 0.78 | 0.433 |
| Region | Europe | topic | 9.90 | 0.042 |
| Region | Europe | topic:age | 2.40 | 0.662 |
| Region | North america | age1 | -1.09 | 0.274 |
| Region | North america | topic | 1.21 | 0.877 |
| Region | North america | topic:age | 2.59 | 0.628 |
| Region | South america | age1 | 0.01 | 0.995 |
| Region | South america | topic | 0.77 | 0.942 |
| Region | South america | topic:age | 0.43 | 0.980 |
| Region | Australasia and pacific | age1 | -0.01 | 0.993 |
| Region | Australasia and pacific | topic | 8.49 | 0.075 |
| Region | Australasia and pacific | topic:age | 6.91 | 0.141 |
| Region | Asia | age1 | 0.01 | 0.995 |
| Region | Asia | topic | 0.00 | 1.000 |
| Region | Asia | topic:age | 0.08 | 0.999 |
| Importance of dispersal in paper | Aim/main focus of paper | age1 | 1.00 | 0.320 |
| Importance of dispersal in paper | Aim/main focus of paper | topic | 8.82 | 0.066 |
| Importance of dispersal in paper | Aim/main focus of paper | topic:age | 2.82 | 0.589 |
| Importance of dispersal in paper | Used in analysis | age1 | 1.78 | 0.075 |
| Importance of dispersal in paper | Used in analysis | topic | 51.46 | 0.000 |
| Importance of dispersal in paper | Used in analysis | topic:age | 9.63 | 0.047 |
| Importance of dispersal in paper | Used in interpretation | age1 | -2.28 | 0.023 |
| Importance of dispersal in paper | Used in interpretation | topic | 31.62 | 0.000 |
| Importance of dispersal in paper | Used in interpretation | topic:age | 3.67 | 0.453 |
| Kind of dispersal knowledge gap | Dispersal distance | age1 | 0.00 | 1.000 |
| Kind of dispersal knowledge gap | Dispersal distance | topic | 0.00 | 1.000 |
| Kind of dispersal knowledge gap | Dispersal distance | topic:age | 0.00 | 1.000 |
| Kind of dispersal knowledge gap | Behaviour | age1 | -0.52 | 0.600 |
| Kind of dispersal knowledge gap | Behaviour | topic | 0.76 | 0.944 |
| Kind of dispersal knowledge gap | Behaviour | topic:age | 2.35 | 0.672 |
| Kind of dispersal knowledge gap | Vegetation-specific dispersal | age1 | -0.53 | 0.600 |
| Kind of dispersal knowledge gap | Vegetation-specific dispersal | topic | 3.00 | 0.558 |
| Kind of dispersal knowledge gap | Vegetation-specific dispersal | topic:age | 3.95 | 0.413 |
| Kind of dispersal knowledge gap | Dispersal rate | age1 | 0.00 | 1.000 |
| Kind of dispersal knowledge gap | Dispersal rate | topic | 0.00 | 1.000 |
| Kind of dispersal knowledge gap | Dispersal rate | topic:age | 0.00 | 1.000 |
| Kind of dispersal knowledge gap | Dispersal success | age1 | -0.34 | 0.734 |
| Kind of dispersal knowledge gap | Dispersal success | topic | 2.12 | 0.713 |
| Kind of dispersal knowledge gap | Dispersal success | topic:age | 2.63 | 0.622 |
| Kind of dispersal knowledge gap | Dispersal vectors | age1 | 0.00 | 0.997 |
| Kind of dispersal knowledge gap | Dispersal vectors | topic | 3.14 | 0.534 |
| Kind of dispersal knowledge gap | Dispersal vectors | topic:age | 0.02 | 1.000 |
| Kind of dispersal knowledge gap | Methodological limitations | age1 | 0.32 | 0.751 |
| Kind of dispersal knowledge gap | Methodological limitations | topic | 2.20 | 0.698 |
| Kind of dispersal knowledge gap | Methodological limitations | topic:age | 0.37 | 0.985 |
| SampleSize |  | age1 | 3.16 | 0.002 |
| SampleSize |  | topic | 10.70 | 0.030 |
| SampleSize |  | topic:age | 14.10 | 0.007 |
| Study duration |  | age1 | -0.28 | 0.778 |
| Study duration |  | topic | 6.69 | 0.153 |
| Study duration |  | topic:age | 1.76 | 0.779 |
| Age of source |  | age1 | -0.82 | 0.415 |
| Age of source |  | topic | 4.94 | 0.293 |
| Age of source |  | topic:age | 3.37 | 0.499 |
