# Supplementary material for: The Trajectory of Dispersal Research in Conservation Biology. Systematic Review
Source: PLoS One. 2014 Apr 17;9(4):e95053. doi: 10.1371/journal.pone.0095053 (PMC3990620; doi:10.1371/journal.pone.0095053)
Supplement: Table S3 — Summary of consistent trends across topics and times. (DOCX) [file pone.0095053.s005.docx]

Table S3. Summary of trends that were consistent across topics and times, and the responses of individual topics. Changes between time periods are indicated in bold type.

|  |  | **Responses specific to each topic** | | | | |
| --- | --- | --- | --- | --- | --- | --- |
| **Response** | **Consistent trends** | **Climate change** | **Invasive species** | **Land planning** | **PVA** | **Restoration** |
| Importance of dispersal in paper | dispersal is used less often in the discussion only | **increased use in analysis** | **increased use in analysis** |  |  | **increased use in analysis** |
| Source | no change over time and no differences among topics |  |  |  |  |  |
| Study type | 1 review for every 6.4 non-review papers on dispersal |  |  | **declining empirical,** majority modelling | majority modelling, few reviews | few modelling studies |
| Use of dispersal knowledge | similar questions addressed in each time period within topic |  |  |  |  |  |
| Method | **genetics increased across all topics.** High use of occupancy, expert opinion and modelling. Low use of new tracking technology |  |  |  | low use of occupancy | high use of occupancy, low use mark-recapture |
| Relevance | relevance of dispersal data high across most topics |  | **increased relevance** |  |  |  |
| Dispersal statistic | no change over time in use of dispersal statistics |  |  |  | high use of dispersal distributions | high use of occupancy, low use dispersal distributions |
| Sample size | no change over time in all topics except climate change | **increased sample size**, and larger than other topics |  |  |  |  |
| Study duration | no change over time and no differences among topics |  |  |  |  |  |
| Age of source | no change over time and no differences among topics |  |  |  |  |  |
| Dispersal knowledge gap present | 46% of studies identify dispersal knowledge as limitation |  |  | highest rates of reporting dispersal as research gap |  | lowest rates of reporting dispersal as research gap |
| Kind of dispersal knowledge gap | key gaps: dispersal distance, dispersal behaviour and vegetation-specific dispersal. No change over time and no differences among topics. |  |  |  |  |  |
| Non-dispersal knowledge gap present | 55% of papers identify non-dispersal limitations |  |  | **increased listing of impediments** (to same level as other topics) |  |  |
| Consequences for study if dispersal data not available | half of studies would have weaker conclusions, one quarter would not be possible |  | higher proportion of studies not possible |  | higher proportion of studies not possible |  |
| Consequences for biodiversity if dispersal data not available | a small proportion of papers for which lack of dispersal data has no consequence |  | highest proportion of papers where knowledge of ecological processes would be limited | high proportion of papers where effectiveness of alternative management strategies unknown | **increased proportion of papers where effectiveness of alternative management strategies unknown** | high proportion of papers where effectiveness of alternative management strategies unknown |
| Taxon | few reptile or amphibian studies. | **studies of plants declined** | insects often studied, **plant studies declined.** | vertebrates often studied, few plant studies | vertebrates often studied, few plant studies | plants most common |
| Biome | majority of studies terrestrial |  | **decline of terrestrial studies** | **decline of terrestrial studies** |  | **decline of terrestrial studies** |
| Region | majority of studies from North America and Europe | under-represented in Australasia-Pacific, and Africa | least commonly addressed in Europe, emphasised in Africa |  |  | most commonly addressed in Europe |
