# Supplementary material for: The Trajectory of Dispersal Research in Conservation Biology. Systematic Review
Source: PLoS One. 2014 Apr 17;9(4):e95053. doi: 10.1371/journal.pone.0095053 (PMC3990620; doi:10.1371/journal.pone.0095053)
Supplement: Table S4 — Number of papers in which dispersal data was gathered using particular methods. (DOCX) [file pone.0095053.s006.docx]

Table S4. Number of papers in which dispersal data was gathered using particular methods.

| **Method of gathering dispersal data** | **Total papers** | **Climate old** | **Climate new** | **Invas-ives old** | **Invas-**  **ives new** | **Plan-ning old** | **Plan-ning new** | **PVA old** | **PVA new** | **Restor-**  **ation old** | **Restor-**  **ation new** |
| --- | --- | --- | --- | --- | --- | --- | --- | --- | --- | --- | --- |
| Habitat occupancy | 94 | 10 | 9 | 21 | 8 | 5 | 8 | 6 | 4 | 10 | 13 |
| Expert opinion | 60 | 8 | 8 | 3 | 4 | 3 | 6 | 5 | 5 | 10 | 8 |
| Modelling | 38 | 2 | 1 | 0 | 2 | 1 | 11 | 10 | 10 | 0 | 1 |
| Measure arrival from known sources | 32 | 2 | 0 | 6 | 9 | 0 | 1 | 0 | 2 | 8 | 4 |
| Mark-recapture | 29 | 1 | 10 | 1 | 4 | 0 | 2 | 0 | 4 | 2 | 5 |
| Review | 29 | 4 | 5 | 4 | 4 | 1 | 4 | 2 | 0 | 4 | 1 |
| Genetics | 28 | 1 | 2 | 0 | 2 | 3 | 3 | 9 | 6 | 2 | 0 |
| Theoretical | 25 | 6 | 3 | 0 | 1 | 2 | 0 | 6 | 7 | 0 | 0 |
| Radio-tracking | 18 | 0 | 3 | 0 | 3 | 2 | 1 | 3 | 5 | 1 | 0 |
| Seed traps | 12 | 1 | 0 | 2 | 2 | 0 | 0 | 1 | 0 | 2 | 4 |
| Direct observation | 11 | 1 | 0 | 3 | 2 | 0 | 1 | 3 | 1 | 0 | 0 |
| Compositional similarity among sites | 7 | 0 | 2 | 0 | 0 | 0 | 1 | 0 | 0 | 2 | 2 |
| Inference based on traits | 7 | 1 | 2 | 1 | 1 | 0 | 0 | 0 | 0 | 0 | 2 |
| Sediment cores | 6 | 3 | 0 | 2 | 0 | 0 | 0 | 0 | 0 | 1 | 0 |
| Pollen or diaspore counts on animals | 6 | 0 | 0 | 2 | 1 | 0 | 0 | 0 | 0 | 2 | 1 |
| Long term monitoring | 5 | 2 | 0 | 2 | 0 | 0 | 0 | 1 | 0 | 0 | 0 |
| Aerial photographs | 3 | 0 | 0 | 0 | 2 | 0 | 0 | 0 | 0 | 0 | 1 |
| Stable isotope analysis | 3 | 0 | 0 | 0 | 0 | 0 | 0 | 0 | 2 | 0 | 1 |
| Estimated arrival dates based on organism growth rate and current size | 2 | 1 | 1 | 0 | 0 | 0 | 0 | 0 | 0 | 0 | 0 |
| GPS tracking | 2 | 0 | 0 | 0 | 0 | 0 | 1 | 0 | 1 | 0 | 0 |
| Inference based on habitat quality | 1 | 0 | 0 | 0 | 0 | 0 | 0 | 0 | 0 | 1 | 0 |
| Landholder questionnaires | 1 | 0 | 0 | 0 | 0 | 0 | 1 | 0 | 0 | 0 | 0 |
| Satellite tracking | 1 | 0 | 0 | 0 | 0 | 0 | 0 | 0 | 1 | 0 | 0 |
| Sediment cores | 1 | 1 | 0 | 0 | 0 | 0 | 0 | 0 | 0 | 0 | 0 |
| Simulation of wind dispersal | 1 | 0 | 0 | 0 | 0 | 0 | 0 | 0 | 0 | 1 | 0 |
| Papers for which method could not be established | 56 | 6 | 4 | 4 | 7 | 1 | 9 | 4 | 5 | 6 | 10 |
